# Supplementary material for: Structure- and computational-aided engineering of an oxidase to produce isoeugenol from a lignin-derived compound
Source: Nat Commun. 2022 Nov 23;13:7195. doi: 10.1038/s41467-022-34912-3 (PMC9684555; doi:10.1038/s41467-022-34912-3)
Supplement: Supplementary file 1 — Supplementary Information [file 41467_2022_34912_MOESM1_ESM.pdf]

Supplementary Information

Structure- and computational-aided engineering of an oxidase to produce isoeugenol from a lignin-derived compound

Yiming Guo[1], Laura Alvigini[2], Milos Trajkovic[1], Lur Alonso-Cotchico[3], Emanuele Monza[3], Simone Savino[1], Ivana Marić[1], Andrea Mattevi[2], Marco W. Fraaije[1*]

[1] Molecular Enzymology Group, University of Groningen, Groningen, the Netherlands

[2] Department of Biology and Biotechnology "Lazzaro Spallanzani", University of Pavia, Pavia, Italy

[3] Zymvol Biomodeling S.L., Barcelona, Spain

Contents

Supplementary Methods 3

Preparative scale conversion of 4-*n*-propylguaiacol 3

Supplementary List of sequence identifiers 4

Supplementary Table 1. 13

Supplementary Table 2. 14

Supplementary Table 3. 15

Supplementary Figure 1. 16

Supplementary Figure 2 17

Supplementary Figure 3. 18

Supplementary Methods

Preparative scale conversion of 4-*n*-propylguaiacol

Preparative conversions were performed using both the purified PROGO as well as whole cells. A reaction volume of 60 mL containing 50 mM KPi pH 7.5, 18.5 mg (0.30 µmol) purified PROGO, 10% (v/v) DMSO, and 0.5 gram (3.0 mmol) 4-*n*-propylguaiacol was added to a 500 mL flask. A 125 mL reaction volume containing 50 mM KPi pH 7.5, PROGO-expressing *E. coli* cells from a 150 mL culture (final OD_{600} = 29), 10% (v/v) DMSO, and 1.27 gram (7.6 mmol) 4-*n*-propylguaiacol was added to another 500 mL flask. Both flasks were placed at 25 °C while shaking (6 x g). Samples were analyzed by HPLC as mentioned before to monitor conversion. After 48 h, both reaction mixtures were extracted with ethyl acetate. The organic layers were washed with water, brine and dried over anhydrous $MgSO_4$. After removal of the solvent under reduced pressure, the residues were purified by chromatography on a silica gel column with hexane-EtOAc (95/5) to afford isoeugenol as a mixture of *E* and *Z* isomers. The PROGO-catalyzed reaction yielded 328 mg (2.0 mmol, 66%, *E*/*Z* = 75/25), while the whole-cells catalyzed reaction afforded 524 mg (3.2 mmol, 42%, *E*/*Z* = 75/25) of isoeugenol as a yellow oil. NMR spectra are shown in Supplementary Figure 3.

^{1}**H NMR** (400 MHz, $CDCl_3$) δ (ppm): ***E*-isomer**: 6.93 – 6.81 (m, 3H, Ar*H*), 6.33 (m, 1H, (C)C*H*), 6.09 (dq, *J* = 15.6, 6.6 Hz, 1H, CHC*H*CH_3), 5.61 (s, 1H, O*H*), 3.90 (s, 1H, OCH_3), 1.87 (dd, *J* = 6.6, 1.3 Hz, 3H, CH_3). ***Z*-isomer**: 6.93 – 6.81 (m, 3H, Ar*H*), 6.33 (m, 1H, (C)C*H*), 5.71 (dq, *J* = 11.7, 7.2 Hz, 1H, CHC*H*CH_3), 5.65 (s, 1H, O*H*), 3.89 (s, 1H, OCH_3), 1.91 (dd, *J* = 7.2, 1.6 Hz, 3H, CH_3).

^{13}**C NMR** (101 MHz, $CDCl_3$) δ (ppm): ***E*-isomer**: 146.52, 144.72, 130.70, 130.63, 123.41, 119.28, 114.32, 107.84, 55.81, 18.32. ***Z*-isomer**: 146.09, 144.24, 130.05, 129.60, 125.13, 122.06, 114.05, 111.38, 55.85, 14.62.

Supplementary List of sequence identifiers

List of sequence identifiers (Uniprot and Uniparc) of the sequences used for the multiple sequence alignment.

query: PDB 5FXD; Q0SBK1 (https://rest.uniprot.org/uniprotkb/Q0SBK1); UPI00020EEC90 (https://rest.uniprot.org/uniparc/UPI00020EEC90); B8M1T5 (https://rest.uniprot.org/uniprotkb/B8M1T5); A4XDV9 (https://rest.uniprot.org/uniprotkb/A4XDV9); Q140Q7 (https://rest.uniprot.org/uniprotkb/Q140Q7); P56216 (https://rest.uniprot.org/uniprotkb/P56216); A7F673 (https://rest.uniprot.org/uniprotkb/A7F673); C7ZCV5 (https://rest.uniprot.org/uniprotkb/C7ZCV5); C7ZAL4 (https://rest.uniprot.org/uniprotkb/C7ZAL4); Q2G889 (https://rest.uniprot.org/uniprotkb/Q2G889); E1TAI0 (https://rest.uniprot.org/uniprotkb/E1TAI0); D5W7A0 (https://rest.uniprot.org/uniprotkb/D5W7A0); B6HN16 (https://rest.uniprot.org/uniprotkb/B6HN16); C7Z853 (https://rest.uniprot.org/uniprotkb/C7Z853); B2JKR1 (https://rest.uniprot.org/uniprotkb/B2JKR1); B6QEH9 (https://rest.uniprot.org/uniprotkb/B6QEH9); B6QKQ3 (https://rest.uniprot.org/uniprotkb/B6QKQ3); E3QPN3 (https://rest.uniprot.org/uniprotkb/E3QPN3); Q2Y8Z3 (https://rest.uniprot.org/uniprotkb/Q2Y8Z3); D8P7G8 (https://rest.uniprot.org/uniprotkb/D8P7G8); Q82WE3 (https://rest.uniprot.org/uniprotkb/Q82WE3); Q5P0I7 (https://rest.uniprot.org/uniprotkb/Q5P0I7); A4XEH8 (https://rest.uniprot.org/uniprotkb/A4XEH8); Q0AFI4 (https://rest.uniprot.org/uniprotkb/Q0AFI4); C4ZM78 (https://rest.uniprot.org/uniprotkb/C4ZM78); P09788 (https://rest.uniprot.org/uniprotkb/P09788); E4U3F6 (https://rest.uniprot.org/uniprotkb/E4U3F6); A1VU06 (https://rest.uniprot.org/uniprotkb/A1VU06); Q5P460 (https://rest.uniprot.org/uniprotkb/Q5P460); Q2G4B1 (https://rest.uniprot.org/uniprotkb/Q2G4B1); B5I6J1 (https://rest.uniprot.org/uniprotkb/B5I6J1); Q9RDU1 (https://rest.uniprot.org/uniprotkb/Q9RDU1); D0J2C9 (https://rest.uniprot.org/uniprotkb/D0J2C9); Q8L385 (https://rest.uniprot.org/uniprotkb/Q8L385); Q2G7T1 (https://rest.uniprot.org/uniprotkb/Q2G7T1); B5I6I6 (https://rest.uniprot.org/uniprotkb/B5I6I6); Q2G426 (https://rest.uniprot.org/uniprotkb/Q2G426); C6JUF6 (https://rest.uniprot.org/uniprotkb/C6JUF6); Q39TS0 (https://rest.uniprot.org/uniprotkb/Q39TS0); UPI00020EEA98 (https://rest.uniprot.org/uniparc/UPI00020EEA98); Q8KP40 (https://rest.uniprot.org/uniprotkb/Q8KP40); B9M8F8 (https://rest.uniprot.org/uniprotkb/B9M8F8); C5P0W2 (https://rest.uniprot.org/uniprotkb/C5P0W2); E4ZZH9 (https://rest.uniprot.org/uniprotkb/E4ZZH9); Q3J8I2 (https://rest.uniprot.org/uniprotkb/Q3J8I2); B6Q5P9 (https://rest.uniprot.org/uniprotkb/B6Q5P9); C8VB34 (https://rest.uniprot.org/uniprotkb/C8VB34); B2VZW1 (https://rest.uniprot.org/uniprotkb/B2VZW1); C3XN60 (https://rest.uniprot.org/uniprotkb/C3XN60); UPI000023DC3E (https://rest.uniprot.org/uniparc/UPI000023DC3E); Q5P8T2 (https://rest.uniprot.org/uniprotkb/Q5P8T2); F0XEU9 (https://rest.uniprot.org/uniprotkb/F0XEU9); C5ZWG6 (https://rest.uniprot.org/uniprotkb/C5ZWG6); C7YSZ5 (https://rest.uniprot.org/uniprotkb/C7YSZ5); Q5P8S5 (https://rest.uniprot.org/uniprotkb/Q5P8S5); A2QTA3 (https://rest.uniprot.org/uniprotkb/A2QTA3); C7ZBY5 (https://rest.uniprot.org/uniprotkb/C7ZBY5); Q0UWK9 (https://rest.uniprot.org/uniprotkb/Q0UWK9); C6E1Z5 (https://rest.uniprot.org/uniprotkb/C6E1Z5); Q090F4 (https://rest.uniprot.org/uniprotkb/Q090F4); B2AM90 (https://rest.uniprot.org/uniprotkb/B2AM90); D5WI51 (https://rest.uniprot.org/uniprotkb/D5WI51); Q39TR9 (https://rest.uniprot.org/uniprotkb/Q39TR9); B9M8F7 (https://rest.uniprot.org/uniprotkb/B9M8F7); A4XDW3 (https://rest.uniprot.org/uniprotkb/A4XDW3); UPI00020EF252 (https://rest.uniprot.org/uniparc/UPI00020EF252); Q4WXH2 (https://rest.uniprot.org/uniprotkb/Q4WXH2); C9SL18 (https://rest.uniprot.org/uniprotkb/C9SL18); B2AS11 (https://rest.uniprot.org/uniprotkb/B2AS11); C7ZJM8 (https://rest.uniprot.org/uniprotkb/C7ZJM8); E3HJC3 (https://rest.uniprot.org/uniprotkb/E3HJC3); B2A9M6 (https://rest.uniprot.org/uniprotkb/B2A9M6); D8K6X1 (https://rest.uniprot.org/uniprotkb/D8K6X1); C8VNI8 (https://rest.uniprot.org/uniprotkb/C8VNI8); C7ZQZ0 (https://rest.uniprot.org/uniprotkb/C7ZQZ0); E0UH34 (https://rest.uniprot.org/uniprotkb/E0UH34); Q026P1 (https://rest.uniprot.org/uniprotkb/Q026P1); B8KJF8 (https://rest.uniprot.org/uniprotkb/B8KJF8); Q82SS0 (https://rest.uniprot.org/uniprotkb/Q82SS0); A8YL09 (https://rest.uniprot.org/uniprotkb/A8YL09); B9ZUK6 (https://rest.uniprot.org/uniprotkb/B9ZUK6); B2AZR7 (https://rest.uniprot.org/uniprotkb/B2AZR7); D2VNJ0 (https://rest.uniprot.org/uniprotkb/D2VNJ0); Q8KP03 (https://rest.uniprot.org/uniprotkb/Q8KP03); D1Z807 (https://rest.uniprot.org/uniprotkb/D1Z807); D2VCG3 (https://rest.uniprot.org/uniprotkb/D2VCG3); Q0AIC5 (https://rest.uniprot.org/uniprotkb/Q0AIC5); B8CNZ5 (https://rest.uniprot.org/uniprotkb/B8CNZ5); A1ZQQ0 (https://rest.uniprot.org/uniprotkb/A1ZQQ0); D2L335 (https://rest.uniprot.org/uniprotkb/D2L335); Q2G891 (https://rest.uniprot.org/uniprotkb/Q2G891); Q47VX6 (https://rest.uniprot.org/uniprotkb/Q47VX6); E4UWZ1 (https://rest.uniprot.org/uniprotkb/E4UWZ1); Q2RPN8 (https://rest.uniprot.org/uniprotkb/Q2RPN8); Q7N7Z9 (https://rest.uniprot.org/uniprotkb/Q7N7Z9); Q15WL0 (https://rest.uniprot.org/uniprotkb/Q15WL0); F4AQB5 (https://rest.uniprot.org/uniprotkb/F4AQB5); D1Z808 (https://rest.uniprot.org/uniprotkb/D1Z808); C9SL19 (https://rest.uniprot.org/uniprotkb/C9SL19); A1WGI0 (https://rest.uniprot.org/uniprotkb/A1WGI0); C5BK15 (https://rest.uniprot.org/uniprotkb/C5BK15); E0QE65 (https://rest.uniprot.org/uniprotkb/E0QE65); UPI0001AEC264 (https://rest.uniprot.org/uniparc/UPI0001AEC264); E3QLZ5 (https://rest.uniprot.org/uniprotkb/E3QLZ5); C9NXE1 (https://rest.uniprot.org/uniprotkb/C9NXE1); C6WR29 (https://rest.uniprot.org/uniprotkb/C6WR29); F2G8P0 (https://rest.uniprot.org/uniprotkb/F2G8P0); B3PBB8

(https://rest.uniprot.org/uniprotkb/B3PBB8); E6XDT8 (https://rest.uniprot.org/uniprotkb/E6XDT8); A8ZVB8 (https://rest.uniprot.org/uniprotkb/A8ZVB8); Q489I0 (https://rest.uniprot.org/uniprotkb/Q489I0); D7B204 (https://rest.uniprot.org/uniprotkb/D7B204); D9V9K8 (https://rest.uniprot.org/uniprotkb/D9V9K8); C8XC31 (https://rest.uniprot.org/uniprotkb/C8XC31); D2PZQ4 (https://rest.uniprot.org/uniprotkb/D2PZQ4); UPI00020F518A (https://rest.uniprot.org/uniparc/UPI00020F518A); D9QS15 (https://rest.uniprot.org/uniprotkb/D9QS15); Q5XQB4 (https://rest.uniprot.org/uniprotkb/Q5XQB4); A4IN49 (https://rest.uniprot.org/uniprotkb/A4IN49); D5MNA0 (https://rest.uniprot.org/uniprotkb/D5MNA0); E6SEL1 (https://rest.uniprot.org/uniprotkb/E6SEL1); F0DKS5 (https://rest.uniprot.org/uniprotkb/F0DKS5); A6TLH8 (https://rest.uniprot.org/uniprotkb/A6TLH8); E1QGP6 (https://rest.uniprot.org/uniprotkb/E1QGP6); A4FNB1 (https://rest.uniprot.org/uniprotkb/A4FNB1); A1HPQ0 (https://rest.uniprot.org/uniprotkb/A1HPQ0); B5ECG4 (https://rest.uniprot.org/uniprotkb/B5ECG4); C5D2K7 (https://rest.uniprot.org/uniprotkb/C5D2K7); B8FLZ3 (https://rest.uniprot.org/uniprotkb/B8FLZ3); Q8L2C3 (https://rest.uniprot.org/uniprotkb/Q8L2C3); B8FVY6 (https://rest.uniprot.org/uniprotkb/B8FVY6); E3ANH6 (https://rest.uniprot.org/uniprotkb/E3ANH6); Q3A0V7 (https://rest.uniprot.org/uniprotkb/Q3A0V7); Q8EML1 (https://rest.uniprot.org/uniprotkb/Q8EML1); Q12XD1 (https://rest.uniprot.org/uniprotkb/Q12XD1); Q9K9B9 (https://rest.uniprot.org/uniprotkb/Q9K9B9); Q9KB02 (https://rest.uniprot.org/uniprotkb/Q9KB02); A9BF95 (https://rest.uniprot.org/uniprotkb/A9BF95); Q3AEZ1 (https://rest.uniprot.org/uniprotkb/Q3AEZ1); C4RD17 (https://rest.uniprot.org/uniprotkb/C4RD17); B0TF00 (https://rest.uniprot.org/uniprotkb/B0TF00); B3E2D9 (https://rest.uniprot.org/uniprotkb/B3E2D9); E2ZBW9 (https://rest.uniprot.org/uniprotkb/E2ZBW9); Q2UKB5 (https://rest.uniprot.org/uniprotkb/Q2UKB5); B8FB80 (https://rest.uniprot.org/uniprotkb/B8FB80); A1HQY6 (https://rest.uniprot.org/uniprotkb/A1HQY6); Q2LWG4 (https://rest.uniprot.org/uniprotkb/Q2LWG4); C9RBT3 (https://rest.uniprot.org/uniprotkb/C9RBT3); A1HLU0 (https://rest.uniprot.org/uniprotkb/A1HLU0); C0WD57 (https://rest.uniprot.org/uniprotkb/C0WD57); E2ZBM8 (https://rest.uniprot.org/uniprotkb/E2ZBM8); D1C2F4 (https://rest.uniprot.org/uniprotkb/D1C2F4); A4IK99 (https://rest.uniprot.org/uniprotkb/A4IK99); B2TIV2 (https://rest.uniprot.org/uniprotkb/B2TIV2); C5DLY6 (https://rest.uniprot.org/uniprotkb/C5DLY6); D5DGL0 (https://rest.uniprot.org/uniprotkb/D5DGL0); F0SW17 (https://rest.uniprot.org/uniprotkb/F0SW17); E3IFQ0 (https://rest.uniprot.org/uniprotkb/E3IFQ0); E5WMB2 (https://rest.uniprot.org/uniprotkb/E5WMB2); D9QS18 (https://rest.uniprot.org/uniprotkb/D9QS18); E8LDR7 (https://rest.uniprot.org/uniprotkb/E8LDR7); A1HPH4 (https://rest.uniprot.org/uniprotkb/A1HPH4); D6XWE2 (https://rest.uniprot.org/uniprotkb/D6XWE2); D8HL37 (https://rest.uniprot.org/uniprotkb/D8HL37); E8T2G0 (https://rest.uniprot.org/uniprotkb/E8T2G0); E4TJR0 (https://rest.uniprot.org/uniprotkb/E4TJR0); A9CZP8 (https://rest.uniprot.org/uniprotkb/A9CZP8); Q3ACK3 (https://rest.uniprot.org/uniprotkb/Q3ACK3); E2Z9N8 (https://rest.uniprot.org/uniprotkb/E2Z9N8); C0WAU6 (https://rest.uniprot.org/uniprotkb/C0WAU6); F0S2X8 (https://rest.uniprot.org/uniprotkb/F0S2X8); A5DDR1 (https://rest.uniprot.org/uniprotkb/A5DDR1); C1PB43 (https://rest.uniprot.org/uniprotkb/C1PB43); D7AFM4 (https://rest.uniprot.org/uniprotkb/D7AFM4); E2ZBS3 (https://rest.uniprot.org/uniprotkb/E2ZBS3); Q1AS83 (https://rest.uniprot.org/uniprotkb/Q1AS83); Q2B5K5 (https://rest.uniprot.org/uniprotkb/Q2B5K5); E8REN4 (https://rest.uniprot.org/uniprotkb/E8REN4); F4FFQ3 (https://rest.uniprot.org/uniprotkb/F4FFQ3); Q5WCX8 (https://rest.uniprot.org/uniprotkb/Q5WCX8); A8MAT1 (https://rest.uniprot.org/uniprotkb/A8MAT1); E5WK13 (https://rest.uniprot.org/uniprotkb/E5WK13); A8V335 (https://rest.uniprot.org/uniprotkb/A8V335); B8FVI5 (https://rest.uniprot.org/uniprotkb/B8FVI5); B9KXS2 (https://rest.uniprot.org/uniprotkb/B9KXS2); D2SGQ1 (https://rest.uniprot.org/uniprotkb/D2SGQ1); E0XUK6 (https://rest.uniprot.org/uniprotkb/E0XUK6); A5G3L8 (https://rest.uniprot.org/uniprotkb/A5G3L8); D5DG79 (https://rest.uniprot.org/uniprotkb/D5DG79); A6CBM8 (https://rest.uniprot.org/uniprotkb/A6CBM8); A1HM32 (https://rest.uniprot.org/uniprotkb/A1HM32); A4FHK5 (https://rest.uniprot.org/uniprotkb/A4FHK5); F4A1J8 (https://rest.uniprot.org/uniprotkb/F4A1J8); P94535 (https://rest.uniprot.org/uniprotkb/P94535); B7GLG2 (https://rest.uniprot.org/uniprotkb/B7GLG2); B0AAX2 (https://rest.uniprot.org/uniprotkb/B0AAX2); F2LWH4 (https://rest.uniprot.org/uniprotkb/F2LWH4); UPI000038E4C0 (https://rest.uniprot.org/uniparc/UPI000038E4C0); D6B4E8 (https://rest.uniprot.org/uniprotkb/D6B4E8); D7CWL8 (https://rest.uniprot.org/uniprotkb/D7CWL8); A5W1Z8 (https://rest.uniprot.org/uniprotkb/A5W1Z8); D5AQ09 (https://rest.uniprot.org/uniprotkb/D5AQ09); D5Q5G6 (https://rest.uniprot.org/uniprotkb/D5Q5G6); B9YDS8 (https://rest.uniprot.org/uniprotkb/B9YDS8); A6LQ70 (https://rest.uniprot.org/uniprotkb/A6LQ70); D2AZJ7 (https://rest.uniprot.org/uniprotkb/D2AZJ7); C0Z926 (https://rest.uniprot.org/uniprotkb/C0Z926); Q2RG44 (https://rest.uniprot.org/uniprotkb/Q2RG44); A3J194 (https://rest.uniprot.org/uniprotkb/A3J194); A1AU26 (https://rest.uniprot.org/uniprotkb/A1AU26); Q6ASD7 (https://rest.uniprot.org/uniprotkb/Q6ASD7); C0QS81 (https://rest.uniprot.org/uniprotkb/C0QS81); D6Z6A3 (https://rest.uniprot.org/uniprotkb/D6Z6A3); F4DP67 (https://rest.uniprot.org/uniprotkb/F4DP67); D4MUZ9 (https://rest.uniprot.org/uniprotkb/D4MUZ9); C4Y691 (https://rest.uniprot.org/uniprotkb/C4Y691); E8R216 (https://rest.uniprot.org/uniprotkb/E8R216); A7HBJ7 (https://rest.uniprot.org/uniprotkb/A7HBJ7); E5VT07 (https://rest.uniprot.org/uniprotkb/E5VT07); O67645 (https://rest.uniprot.org/uniprotkb/O67645); F4LT71 (https://rest.uniprot.org/uniprotkb/F4LT71); E8LBD8 (https://rest.uniprot.org/uniprotkb/E8LBD8); D1CEH5 (https://rest.uniprot.org/uniprotkb/D1CEH5); Q6CWU6 (https://rest.uniprot.org/uniprotkb/Q6CWU6); B1R055 (https://rest.uniprot.org/uniprotkb/B1R055);

A3VYL0 (https://rest.uniprot.org/uniprotkb/A3VYL0); UPI00020EB24E (https://rest.uniprot.org/uniparc/UPI00020EB24E); E0QE63 (https://rest.uniprot.org/uniprotkb/E0QE63); C5CH77 (https://rest.uniprot.org/uniprotkb/C5CH77); A8F7W4 (https://rest.uniprot.org/uniprotkb/A8F7W4); D9TQ03 (https://rest.uniprot.org/uniprotkb/D9TQ03); UPI0001E896DA (https://rest.uniprot.org/uniparc/UPI0001E896DA); D0LH07 (https://rest.uniprot.org/uniprotkb/D0LH07); D8GN49 (https://rest.uniprot.org/uniprotkb/D8GN49); D9VSN4 (https://rest.uniprot.org/uniprotkb/D9VSN4); F0KCV8 (https://rest.uniprot.org/uniprotkb/F0KCV8); A8FDZ7 (https://rest.uniprot.org/uniprotkb/A8FDZ7); B4UB81 (https://rest.uniprot.org/uniprotkb/B4UB81); A3VI11 (https://rest.uniprot.org/uniprotkb/A3VI11); Q0W3T1 (https://rest.uniprot.org/uniprotkb/Q0W3T1); E8LBD6 (https://rest.uniprot.org/uniprotkb/E8LBD6); D8EZ09 (https://rest.uniprot.org/uniprotkb/D8EZ09); D2PNN5 (https://rest.uniprot.org/uniprotkb/D2PNN5); A8S3I3 (https://rest.uniprot.org/uniprotkb/A8S3I3); Q0F8W0 (https://rest.uniprot.org/uniprotkb/Q0F8W0); C6J4C4 (https://rest.uniprot.org/uniprotkb/C6J4C4); D7AYE2 (https://rest.uniprot.org/uniprotkb/D7AYE2); D1YV81 (https://rest.uniprot.org/uniprotkb/D1YV81); D3M1I0 (https://rest.uniprot.org/uniprotkb/D3M1I0); D6Y8G3 (https://rest.uniprot.org/uniprotkb/D6Y8G3); A5UYL8 (https://rest.uniprot.org/uniprotkb/A5UYL8); E7M3I8 (https://rest.uniprot.org/uniprotkb/E7M3I8); C7ZEQ0 (https://rest.uniprot.org/uniprotkb/C7ZEQ0); A1HLV1 (https://rest.uniprot.org/uniprotkb/A1HLV1); A4FGI5 (https://rest.uniprot.org/uniprotkb/A4FGI5); Q1PW53 (https://rest.uniprot.org/uniprotkb/Q1PW53); B9M1Q0 (https://rest.uniprot.org/uniprotkb/B9M1Q0); A9WM09 (https://rest.uniprot.org/uniprotkb/A9WM09); C6PWC1 (https://rest.uniprot.org/uniprotkb/C6PWC1); A1W221 (https://rest.uniprot.org/uniprotkb/A1W221); E6MHP5 (https://rest.uniprot.org/uniprotkb/E6MHP5); A6G9M1 (https://rest.uniprot.org/uniprotkb/A6G9M1); F3NF70 (https://rest.uniprot.org/uniprotkb/F3NF70); E5BJ17 (https://rest.uniprot.org/uniprotkb/E5BJ17); E5WDS6 (https://rest.uniprot.org/uniprotkb/E5WDS6); B2JGF7 (https://rest.uniprot.org/uniprotkb/B2JGF7); A0PZ91 (https://rest.uniprot.org/uniprotkb/A0PZ91); C0GH85 (https://rest.uniprot.org/uniprotkb/C0GH85); E6R2K7 (https://rest.uniprot.org/uniprotkb/E6R2K7); D3DI81 (https://rest.uniprot.org/uniprotkb/D3DI81); A5N943 (https://rest.uniprot.org/uniprotkb/A5N943); D1CDS6 (https://rest.uniprot.org/uniprotkb/D1CDS6); C1IB77 (https://rest.uniprot.org/uniprotkb/C1IB77); C9Z155 (https://rest.uniprot.org/uniprotkb/C9Z155); D3LT57 (https://rest.uniprot.org/uniprotkb/D3LT57); B0TBD2 (https://rest.uniprot.org/uniprotkb/B0TBD2); C0QK91 (https://rest.uniprot.org/uniprotkb/C0QK91); C8WT22 (https://rest.uniprot.org/uniprotkb/C8WT22); D0B619 (https://rest.uniprot.org/uniprotkb/D0B619); B5YJU5 (https://rest.uniprot.org/uniprotkb/B5YJU5); E0MJ74 (https://rest.uniprot.org/uniprotkb/E0MJ74); Q0VTE7 (https://rest.uniprot.org/uniprotkb/Q0VTE7); D3LWV2 (https://rest.uniprot.org/uniprotkb/D3LWV2); Q67QR5 (https://rest.uniprot.org/uniprotkb/Q67QR5); Q8XNM0 (https://rest.uniprot.org/uniprotkb/Q8XNM0); Q1QCC4 (https://rest.uniprot.org/uniprotkb/Q1QCC4); C7MP87 (https://rest.uniprot.org/uniprotkb/C7MP87); A0LFN1 (https://rest.uniprot.org/uniprotkb/A0LFN1); Q896M6 (https://rest.uniprot.org/uniprotkb/Q896M6); UPI00020A9C22 (https://rest.uniprot.org/uniparc/UPI00020A9C22); E5WP45 (https://rest.uniprot.org/uniprotkb/E5WP45); E8SFV8 (https://rest.uniprot.org/uniprotkb/E8SFV8); A4VGK4 (https://rest.uniprot.org/uniprotkb/A4VGK4); D1ADK6 (https://rest.uniprot.org/uniprotkb/D1ADK6); UPI00020EB47D (https://rest.uniprot.org/uniparc/UPI00020EB47D); E2Z9D7 (https://rest.uniprot.org/uniprotkb/E2Z9D7); E0HZZ9 (https://rest.uniprot.org/uniprotkb/E0HZZ9); D3Q9U4 (https://rest.uniprot.org/uniprotkb/D3Q9U4); B8NUP7 (https://rest.uniprot.org/uniprotkb/B8NUP7); F4PFD0 (https://rest.uniprot.org/uniprotkb/F4PFD0); A4J1G4 (https://rest.uniprot.org/uniprotkb/A4J1G4); C6RQA3 (https://rest.uniprot.org/uniprotkb/C6RQA3); D0IV64 (https://rest.uniprot.org/uniprotkb/D0IV64); D5WU23 (https://rest.uniprot.org/uniprotkb/D5WU23); B1Z2H3 (https://rest.uniprot.org/uniprotkb/B1Z2H3); F1A1D2 (https://rest.uniprot.org/uniprotkb/F1A1D2); E1YKU2 (https://rest.uniprot.org/uniprotkb/E1YKU2); C0GHE6 (https://rest.uniprot.org/uniprotkb/C0GHE6); Q01H57 (https://rest.uniprot.org/uniprotkb/Q01H57); D7GNZ6 (https://rest.uniprot.org/uniprotkb/D7GNZ6); A5I0K1 (https://rest.uniprot.org/uniprotkb/A5I0K1); F4GIU0 (https://rest.uniprot.org/uniprotkb/F4GIU0); C9LUX5 (https://rest.uniprot.org/uniprotkb/C9LUX5); A3JQ06 (https://rest.uniprot.org/uniprotkb/A3JQ06); A0K4R1 (https://rest.uniprot.org/uniprotkb/A0K4R1); A6TKF5 (https://rest.uniprot.org/uniprotkb/A6TKF5); B8FL39 (https://rest.uniprot.org/uniprotkb/B8FL39); C5DWB3 (https://rest.uniprot.org/uniprotkb/C5DWB3); F0DHX5 (https://rest.uniprot.org/uniprotkb/F0DHX5); B5GSM1 (https://rest.uniprot.org/uniprotkb/B5GSM1); A9BQK9 (https://rest.uniprot.org/uniprotkb/A9BQK9); B5Z6N1 (https://rest.uniprot.org/uniprotkb/B5Z6N1); D2RLY3 (https://rest.uniprot.org/uniprotkb/D2RLY3); E1UML4 (https://rest.uniprot.org/uniprotkb/E1UML4); E5Y4N5 (https://rest.uniprot.org/uniprotkb/E5Y4N5); E7R8E7 (https://rest.uniprot.org/uniprotkb/E7R8E7); D6TG87 (https://rest.uniprot.org/uniprotkb/D6TG87); B2HZU3 (https://rest.uniprot.org/uniprotkb/B2HZU3); F2R4U7 (https://rest.uniprot.org/uniprotkb/F2R4U7); Q5WFZ5 (https://rest.uniprot.org/uniprotkb/Q5WFZ5); E3E0W0 (https://rest.uniprot.org/uniprotkb/E3E0W0); E7REH2 (https://rest.uniprot.org/uniprotkb/E7REH2); B6HKH1 (https://rest.uniprot.org/uniprotkb/B6HKH1); D1XPY3 (https://rest.uniprot.org/uniprotkb/D1XPY3); Q08SR9 (https://rest.uniprot.org/uniprotkb/Q08SR9); C9LRK5 (https://rest.uniprot.org/uniprotkb/C9LRK5); A5D027 (https://rest.uniprot.org/uniprotkb/A5D027); C5DQS7 (https://rest.uniprot.org/uniprotkb/C5DQS7); Q02A73 (https://rest.uniprot.org/uniprotkb/Q02A73); A5WCY2 (https://rest.uniprot.org/uniprotkb/A5WCY2); D7AJ54 (https://rest.uniprot.org/uniprotkb/D7AJ54);

UPI00020F4ECE (https://rest.uniprot.org/uniparc/UPI00020F4ECE); A8UYD8 (https://rest.uniprot.org/uniprotkb/A8UYD8); Q0FGU2 (https://rest.uniprot.org/uniprotkb/Q0FGU2); D6EWE2 (https://rest.uniprot.org/uniprotkb/D6EWE2); UPI00020A97F8 (https://rest.uniprot.org/uniparc/UPI00020A97F8); E3H969 (https://rest.uniprot.org/uniprotkb/E3H969); E5BGD0 (https://rest.uniprot.org/uniprotkb/E5BGD0); UPI0001745AB9 (https://rest.uniprot.org/uniparc/UPI0001745AB9); Q65NS5 (https://rest.uniprot.org/uniprotkb/Q65NS5); C6WL15 (https://rest.uniprot.org/uniprotkb/C6WL15); F3YYV8 (https://rest.uniprot.org/uniprotkb/F3YYV8); A2QXM7 (https://rest.uniprot.org/uniprotkb/A2QXM7); E5VQZ3 (https://rest.uniprot.org/uniprotkb/E5VQZ3); E6SBM7 (https://rest.uniprot.org/uniprotkb/E6SBM7); Q1H3E6 (https://rest.uniprot.org/uniprotkb/Q1H3E6); A7F6C9 (https://rest.uniprot.org/uniprotkb/A7F6C9); F2NGA8 (https://rest.uniprot.org/uniprotkb/F2NGA8); D5RKV0 (https://rest.uniprot.org/uniprotkb/D5RKV0); A6FQU9 (https://rest.uniprot.org/uniprotkb/A6FQU9); F0QV22 (https://rest.uniprot.org/uniprotkb/F0QV22); D7BXM7 (https://rest.uniprot.org/uniprotkb/D7BXM7); B4U7V1 (https://rest.uniprot.org/uniprotkb/B4U7V1); Q6APH3 (https://rest.uniprot.org/uniprotkb/Q6APH3); A8I1S6 (https://rest.uniprot.org/uniprotkb/A8I1S6); F4D1M2 (https://rest.uniprot.org/uniprotkb/F4D1M2); B4VCB4 (https://rest.uniprot.org/uniprotkb/B4VCB4); D8GSN8 (https://rest.uniprot.org/uniprotkb/D8GSN8); A3GEX0 (https://rest.uniprot.org/uniprotkb/A3GEX0); D8GKN7 (https://rest.uniprot.org/uniprotkb/D8GKN7); A6DAA1 (https://rest.uniprot.org/uniprotkb/A6DAA1); A0Y9R7 (https://rest.uniprot.org/uniprotkb/A0Y9R7); B8FVY5 (https://rest.uniprot.org/uniprotkb/B8FVY5); A5GC40 (https://rest.uniprot.org/uniprotkb/A5GC40); F0JFZ0 (https://rest.uniprot.org/uniprotkb/F0JFZ0); A7NPI3 (https://rest.uniprot.org/uniprotkb/A7NPI3); B3CZV3 (https://rest.uniprot.org/uniprotkb/B3CZV3); E6UXT6 (https://rest.uniprot.org/uniprotkb/E6UXT6); D7E5U6 (https://rest.uniprot.org/uniprotkb/D7E5U6); B5KE87 (https://rest.uniprot.org/uniprotkb/B5KE87); F4C839 (https://rest.uniprot.org/uniprotkb/F4C839); UPI0001CBC0A1 (https://rest.uniprot.org/uniparc/UPI0001CBC0A1); E5Y531 (https://rest.uniprot.org/uniprotkb/E5Y531); E8T907 (https://rest.uniprot.org/uniprotkb/E8T907); C0QCR3 (https://rest.uniprot.org/uniprotkb/C0QCR3); D5WUI7 (https://rest.uniprot.org/uniprotkb/D5WUI7); E5VT15 (https://rest.uniprot.org/uniprotkb/E5VT15); D5BRS2 (https://rest.uniprot.org/uniprotkb/D5BRS2); B5ZHH0 (https://rest.uniprot.org/uniprotkb/B5ZHH0); B4UJI8 (https://rest.uniprot.org/uniprotkb/B4UJI8); C6XJB2 (https://rest.uniprot.org/uniprotkb/C6XJB2); O29450 (https://rest.uniprot.org/uniprotkb/O29450); A9KNU3 (https://rest.uniprot.org/uniprotkb/A9KNU3); D3BR29 (https://rest.uniprot.org/uniprotkb/D3BR29); B2VAF0 (https://rest.uniprot.org/uniprotkb/B2VAF0); Q11KF9 (https://rest.uniprot.org/uniprotkb/Q11KF9); A3TZC9 (https://rest.uniprot.org/uniprotkb/A3TZC9); E7RKX4 (https://rest.uniprot.org/uniprotkb/E7RKX4); Q30WB3 (https://rest.uniprot.org/uniprotkb/Q30WB3); Q2RMG2 (https://rest.uniprot.org/uniprotkb/Q2RMG2); D8GJQ6 (https://rest.uniprot.org/uniprotkb/D8GJQ6); D8PKI0 (https://rest.uniprot.org/uniprotkb/D8PKI0); B9NM88 (https://rest.uniprot.org/uniprotkb/B9NM88); D6X6G5 (https://rest.uniprot.org/uniprotkb/D6X6G5); A8TJC4 (https://rest.uniprot.org/uniprotkb/A8TJC4); A5FYI8 (https://rest.uniprot.org/uniprotkb/A5FYI8); F0DKC7 (https://rest.uniprot.org/uniprotkb/F0DKC7); B2TJE6 (https://rest.uniprot.org/uniprotkb/B2TJE6); C4LIW4 (https://rest.uniprot.org/uniprotkb/C4LIW4); C6BYM1 (https://rest.uniprot.org/uniprotkb/C6BYM1); A5G1K9 (https://rest.uniprot.org/uniprotkb/A5G1K9); B8DMG2 (https://rest.uniprot.org/uniprotkb/B8DMG2); D9WS76 (https://rest.uniprot.org/uniprotkb/D9WS76); UPI00016C5353 (https://rest.uniprot.org/uniparc/UPI00016C5353); Q1QDR1 (https://rest.uniprot.org/uniprotkb/Q1QDR1); C1DBZ1 (https://rest.uniprot.org/uniprotkb/C1DBZ1); A3KAL7 (https://rest.uniprot.org/uniprotkb/A3KAL7); D0D3D4 (https://rest.uniprot.org/uniprotkb/D0D3D4); B2HIT1 (https://rest.uniprot.org/uniprotkb/B2HIT1); E6PR90 (https://rest.uniprot.org/uniprotkb/E6PR90); A3X5J3 (https://rest.uniprot.org/uniprotkb/A3X5J3); A9FR03 (https://rest.uniprot.org/uniprotkb/A9FR03); Q55BQ4 (https://rest.uniprot.org/uniprotkb/Q55BQ4); D6TRA0 (https://rest.uniprot.org/uniprotkb/D6TRA0); A5UTQ1 (https://rest.uniprot.org/uniprotkb/A5UTQ1); D9WKH9 (https://rest.uniprot.org/uniprotkb/D9WKH9); A0LF90 (https://rest.uniprot.org/uniprotkb/A0LF90); B9XM77 (https://rest.uniprot.org/uniprotkb/B9XM77); B0MCR1 (https://rest.uniprot.org/uniprotkb/B0MCR1); Q7P867 (https://rest.uniprot.org/uniprotkb/Q7P867); D5ZZE2 (https://rest.uniprot.org/uniprotkb/D5ZZE2); D8F3M0 (https://rest.uniprot.org/uniprotkb/D8F3M0); C1DK29 (https://rest.uniprot.org/uniprotkb/C1DK29); F2U5K6 (https://rest.uniprot.org/uniprotkb/F2U5K6); B8NJ50 (https://rest.uniprot.org/uniprotkb/B8NJ50); D9XCJ8 (https://rest.uniprot.org/uniprotkb/D9XCJ8); C5T8Q8 (https://rest.uniprot.org/uniprotkb/C5T8Q8); A9CJZ9 (https://rest.uniprot.org/uniprotkb/A9CJZ9); E1K284 (https://rest.uniprot.org/uniprotkb/E1K284); Q2JI05 (https://rest.uniprot.org/uniprotkb/Q2JI05); D3EK88 (https://rest.uniprot.org/uniprotkb/D3EK88); A1VIB7 (https://rest.uniprot.org/uniprotkb/A1VIB7); B1QSI4 (https://rest.uniprot.org/uniprotkb/B1QSI4); A1CJF1 (https://rest.uniprot.org/uniprotkb/A1CJF1); B6K538 (https://rest.uniprot.org/uniprotkb/B6K538); C3X2S0 (https://rest.uniprot.org/uniprotkb/C3X2S0); A2QY53 (https://rest.uniprot.org/uniprotkb/A2QY53); C4R697 (https://rest.uniprot.org/uniprotkb/C4R697); Q5KYD7 (https://rest.uniprot.org/uniprotkb/Q5KYD7); C7PW09 (https://rest.uniprot.org/uniprotkb/C7PW09); A6DXV5 (https://rest.uniprot.org/uniprotkb/A6DXV5); F2IIF5 (https://rest.uniprot.org/uniprotkb/F2IIF5); D3L5G9 (https://rest.uniprot.org/uniprotkb/D3L5G9); B9JAK2 (https://rest.uniprot.org/uniprotkb/B9JAK2); C7MRI9 (https://rest.uniprot.org/uniprotkb/C7MRI9); D2RNB3 (https://rest.uniprot.org/uniprotkb/D2RNB3); Q5WAP2

(https://rest.uniprot.org/uniprotkb/Q5WAP2); D0D8T0 (https://rest.uniprot.org/uniprotkb/D0D8T0); D9VY89 (https://rest.uniprot.org/uniprotkb/D9VY89); E3GDK6 (https://rest.uniprot.org/uniprotkb/E3GDK6); Q0CC46 (https://rest.uniprot.org/uniprotkb/Q0CC46); D9YHH6 (https://rest.uniprot.org/uniprotkb/D9YHH6); E8RJS9 (https://rest.uniprot.org/uniprotkb/E8RJS9); B6WWC5 (https://rest.uniprot.org/uniprotkb/B6WWC5); E4RLM5 (https://rest.uniprot.org/uniprotkb/E4RLM5); Q0FL57 (https://rest.uniprot.org/uniprotkb/Q0FL57); Q4WAU8 (https://rest.uniprot.org/uniprotkb/Q4WAU8); C4XJ74 (https://rest.uniprot.org/uniprotkb/C4XJ74); D4CQM2 (https://rest.uniprot.org/uniprotkb/D4CQM2); B6QUW7 (https://rest.uniprot.org/uniprotkb/B6QUW7); F2J064 (https://rest.uniprot.org/uniprotkb/F2J064); D3LU73 (https://rest.uniprot.org/uniprotkb/D3LU73); Q8DIG3 (https://rest.uniprot.org/uniprotkb/Q8DIG3); F3Z141 (https://rest.uniprot.org/uniprotkb/F3Z141); A4X1K8 (https://rest.uniprot.org/uniprotkb/A4X1K8); D4MUV9 (https://rest.uniprot.org/uniprotkb/D4MUV9); B9L9B2 (https://rest.uniprot.org/uniprotkb/B9L9B2); Q1LJ79 (https://rest.uniprot.org/uniprotkb/Q1LJ79); F2KMN8 (https://rest.uniprot.org/uniprotkb/F2KMN8); Q6JWT4 (https://rest.uniprot.org/uniprotkb/Q6JWT4); A8TVR6 (https://rest.uniprot.org/uniprotkb/A8TVR6); D3LVD2 (https://rest.uniprot.org/uniprotkb/D3LVD2); B5ZS50 (https://rest.uniprot.org/uniprotkb/B5ZS50); Q4WWR7 (https://rest.uniprot.org/uniprotkb/Q4WWR7); F4GCQ3 (https://rest.uniprot.org/uniprotkb/F4GCQ3); C8VE07 (https://rest.uniprot.org/uniprotkb/C8VE07); C1D5D7 (https://rest.uniprot.org/uniprotkb/C1D5D7); B1J7V4 (https://rest.uniprot.org/uniprotkb/B1J7V4); C5EN78 (https://rest.uniprot.org/uniprotkb/C5EN78); Q1D857 (https://rest.uniprot.org/uniprotkb/Q1D857); E0MQ68 (https://rest.uniprot.org/uniprotkb/E0MQ68); D2S5T0 (https://rest.uniprot.org/uniprotkb/D2S5T0); Q2LXM8 (https://rest.uniprot.org/uniprotkb/Q2LXM8); C4XRF2 (https://rest.uniprot.org/uniprotkb/C4XRF2); B5EIV4 (https://rest.uniprot.org/uniprotkb/B5EIV4); A5D125 (https://rest.uniprot.org/uniprotkb/A5D125); C5EX94 (https://rest.uniprot.org/uniprotkb/C5EX94); B3RIV9 (https://rest.uniprot.org/uniprotkb/B3RIV9); C5ME72 (https://rest.uniprot.org/uniprotkb/C5ME72); P32891 (https://rest.uniprot.org/uniprotkb/P32891); Q12HB6 (https://rest.uniprot.org/uniprotkb/Q12HB6); Q89RT4 (https://rest.uniprot.org/uniprotkb/Q89RT4); D5WQS6 (https://rest.uniprot.org/uniprotkb/D5WQS6); C0QKR7 (https://rest.uniprot.org/uniprotkb/C0QKR7); B2W4L9 (https://rest.uniprot.org/uniprotkb/B2W4L9); UPI0001DE95D0 (https://rest.uniprot.org/uniparc/UPI0001DE95D0); C1DFQ8 (https://rest.uniprot.org/uniprotkb/C1DFQ8); E3CYX4 (https://rest.uniprot.org/uniprotkb/E3CYX4); F3WWY8 (https://rest.uniprot.org/uniprotkb/F3WWY8); B2T8P7 (https://rest.uniprot.org/uniprotkb/B2T8P7); A1TIC0 (https://rest.uniprot.org/uniprotkb/A1TIC0); A1WRY1 (https://rest.uniprot.org/uniprotkb/A1WRY1); Q317B3 (https://rest.uniprot.org/uniprotkb/Q317B3); D5GZR9 (https://rest.uniprot.org/uniprotkb/D5GZR9); UPI00020CCD1C (https://rest.uniprot.org/uniparc/UPI00020CCD1C); A6GX56 (https://rest.uniprot.org/uniprotkb/A6GX56); A2QZV2 (https://rest.uniprot.org/uniprotkb/A2QZV2); D2R6L8 (https://rest.uniprot.org/uniprotkb/D2R6L8); C9YCE6 (https://rest.uniprot.org/uniprotkb/C9YCE6); B6BA99 (https://rest.uniprot.org/uniprotkb/B6BA99); B8H7H9 (https://rest.uniprot.org/uniprotkb/B8H7H9); B2AUQ3 (https://rest.uniprot.org/uniprotkb/B2AUQ3); D5XEL5 (https://rest.uniprot.org/uniprotkb/D5XEL5); UPI00020A9B08 (https://rest.uniprot.org/uniparc/UPI00020A9B08); E7C867 (https://rest.uniprot.org/uniprotkb/E7C867); D8HQG5 (https://rest.uniprot.org/uniprotkb/D8HQG5); B8DRX6 (https://rest.uniprot.org/uniprotkb/B8DRX6); B4CTR6 (https://rest.uniprot.org/uniprotkb/B4CTR6); E8WCR5 (https://rest.uniprot.org/uniprotkb/E8WCR5); Q2W2Y6 (https://rest.uniprot.org/uniprotkb/Q2W2Y6); C9SC79 (https://rest.uniprot.org/uniprotkb/C9SC79); B8GTM3 (https://rest.uniprot.org/uniprotkb/B8GTM3); A4RLF8 (https://rest.uniprot.org/uniprotkb/A4RLF8); B8J2X1 (https://rest.uniprot.org/uniprotkb/B8J2X1); B8EKA2 (https://rest.uniprot.org/uniprotkb/B8EKA2); E3BDK1 (https://rest.uniprot.org/uniprotkb/E3BDK1); C5PIU3 (https://rest.uniprot.org/uniprotkb/C5PIU3); A1RU75 (https://rest.uniprot.org/uniprotkb/A1RU75); A6Q1U6 (https://rest.uniprot.org/uniprotkb/A6Q1U6); C1PFA1 (https://rest.uniprot.org/uniprotkb/C1PFA1); UPI00020EF8F7 (https://rest.uniprot.org/uniparc/UPI00020EF8F7); C3JLG4 (https://rest.uniprot.org/uniprotkb/C3JLG4); A1R3A1 (https://rest.uniprot.org/uniprotkb/A1R3A1); C2BEA0 (https://rest.uniprot.org/uniprotkb/C2BEA0); Q2BNT4 (https://rest.uniprot.org/uniprotkb/Q2BNT4); E2CKC1 (https://rest.uniprot.org/uniprotkb/E2CKC1); D9VFB4 (https://rest.uniprot.org/uniprotkb/D9VFB4); A4EQA9 (https://rest.uniprot.org/uniprotkb/A4EQA9); B8N175 (https://rest.uniprot.org/uniprotkb/B8N175); A3GI83 (https://rest.uniprot.org/uniprotkb/A3GI83); D0CV03 (https://rest.uniprot.org/uniprotkb/D0CV03); Q0CWQ1 (https://rest.uniprot.org/uniprotkb/Q0CWQ1); Q4FNZ0 (https://rest.uniprot.org/uniprotkb/Q4FNZ0); Q1YH40 (https://rest.uniprot.org/uniprotkb/Q1YH40); A9DBH8 (https://rest.uniprot.org/uniprotkb/A9DBH8); C3XG63 (https://rest.uniprot.org/uniprotkb/C3XG63); E1IHB4 (https://rest.uniprot.org/uniprotkb/E1IHB4); A4G4E3 (https://rest.uniprot.org/uniprotkb/A4G4E3); A3SH73 (https://rest.uniprot.org/uniprotkb/A3SH73); E3IJ53 (https://rest.uniprot.org/uniprotkb/E3IJ53); D8GJ22 (https://rest.uniprot.org/uniprotkb/D8GJ22); D3PY05 (https://rest.uniprot.org/uniprotkb/D3PY05); D4J7S8 (https://rest.uniprot.org/uniprotkb/D4J7S8); B2JK37 (https://rest.uniprot.org/uniprotkb/B2JK37); F3YWL8 (https://rest.uniprot.org/uniprotkb/F3YWL8); Q11LF7 (https://rest.uniprot.org/uniprotkb/Q11LF7); C7N5W3 (https://rest.uniprot.org/uniprotkb/C7N5W3); Q6BSH7 (https://rest.uniprot.org/uniprotkb/Q6BSH7); F2F6H6 (https://rest.uniprot.org/uniprotkb/F2F6H6); D9X930 (https://rest.uniprot.org/uniprotkb/D9X930); A7I3P7 (https://rest.uniprot.org/uniprotkb/A7I3P7); E2ZAY0 (https://rest.uniprot.org/uniprotkb/E2ZAY0);

Q1NAW5 (https://rest.uniprot.org/uniprotkb/Q1NAW5); B7JEZ4 (https://rest.uniprot.org/uniprotkb/B7JEZ4); D9UWA7 (https://rest.uniprot.org/uniprotkb/D9UWA7); A1WDW1 (https://rest.uniprot.org/uniprotkb/A1WDW1); B7R5X5 (https://rest.uniprot.org/uniprotkb/B7R5X5); Q148K4 (https://rest.uniprot.org/uniprotkb/Q148K4); A6TUJ8 (https://rest.uniprot.org/uniprotkb/A6TUJ8); F0JIF5 (https://rest.uniprot.org/uniprotkb/F0JIF5); C3XMX9 (https://rest.uniprot.org/uniprotkb/C3XMX9); A9EJ18 (https://rest.uniprot.org/uniprotkb/A9EJ18); F2L3M2 (https://rest.uniprot.org/uniprotkb/F2L3M2); E6SHX0 (https://rest.uniprot.org/uniprotkb/E6SHX0); Q6BMB0 (https://rest.uniprot.org/uniprotkb/Q6BMB0); C0QBU7 (https://rest.uniprot.org/uniprotkb/C0QBU7); C3N9Z0 (https://rest.uniprot.org/uniprotkb/C3N9Z0); E8LBD7 (https://rest.uniprot.org/uniprotkb/E8LBD7); D3NXA0 (https://rest.uniprot.org/uniprotkb/D3NXA0); B9W6L4 (https://rest.uniprot.org/uniprotkb/B9W6L4); D2L2G6 (https://rest.uniprot.org/uniprotkb/D2L2G6); F4CND6 (https://rest.uniprot.org/uniprotkb/F4CND6); A1KB42 (https://rest.uniprot.org/uniprotkb/A1KB42); Q0G3W8 (https://rest.uniprot.org/uniprotkb/Q0G3W8); A4IT27 (https://rest.uniprot.org/uniprotkb/A4IT27); E2CAI9 (https://rest.uniprot.org/uniprotkb/E2CAI9); A3PHU0 (https://rest.uniprot.org/uniprotkb/A3PHU0); C7D8M7 (https://rest.uniprot.org/uniprotkb/C7D8M7); A8PU95 (https://rest.uniprot.org/uniprotkb/A8PU95); Q46XI5 (https://rest.uniprot.org/uniprotkb/Q46XI5); Q2SLH3 (https://rest.uniprot.org/uniprotkb/Q2SLH3); E3HEJ8 (https://rest.uniprot.org/uniprotkb/E3HEJ8); B8HU29 (https://rest.uniprot.org/uniprotkb/B8HU29); D1B3D8 (https://rest.uniprot.org/uniprotkb/D1B3D8); Q86WU2-2 (https://rest.uniprot.org/uniprotkb/Q86WU2-2); C7FPB0 (https://rest.uniprot.org/uniprotkb/C7FPB0); C7FPF8 (https://rest.uniprot.org/uniprotkb/C7FPF8); E3Q756 (https://rest.uniprot.org/uniprotkb/E3Q756); UPI0001DE68EE (https://rest.uniprot.org/uniparc/UPI0001DE68EE); C4XWB1 (https://rest.uniprot.org/uniprotkb/C4XWB1); A4VGN6 (https://rest.uniprot.org/uniprotkb/A4VGN6); Q7VHU4 (https://rest.uniprot.org/uniprotkb/Q7VHU4); Q0W2Q0 (https://rest.uniprot.org/uniprotkb/Q0W2Q0); Q1GJF0 (https://rest.uniprot.org/uniprotkb/Q1GJF0); E8TNA9 (https://rest.uniprot.org/uniprotkb/E8TNA9); C4JZH6 (https://rest.uniprot.org/uniprotkb/C4JZH6); C4JE22 (https://rest.uniprot.org/uniprotkb/C4JE22); C8PJB8 (https://rest.uniprot.org/uniprotkb/C8PJB8); D0LRK9 (https://rest.uniprot.org/uniprotkb/D0LRK9); B0PGS1 (https://rest.uniprot.org/uniprotkb/B0PGS1); C5SZX2 (https://rest.uniprot.org/uniprotkb/C5SZX2); D5E8F7 (https://rest.uniprot.org/uniprotkb/D5E8F7); Q12V64 (https://rest.uniprot.org/uniprotkb/Q12V64); UPI000023F637 (https://rest.uniprot.org/uniparc/UPI000023F637); E3Q3K6 (https://rest.uniprot.org/uniprotkb/E3Q3K6); F3ED31 (https://rest.uniprot.org/uniprotkb/F3ED31); A3PKQ2 (https://rest.uniprot.org/uniprotkb/A3PKQ2); B3QID7 (https://rest.uniprot.org/uniprotkb/B3QID7); A8IGF7 (https://rest.uniprot.org/uniprotkb/A8IGF7); F2I320 (https://rest.uniprot.org/uniprotkb/F2I320); D7B762 (https://rest.uniprot.org/uniprotkb/D7B762); Q50685 (https://rest.uniprot.org/uniprotkb/Q50685); Q2H4F5 (https://rest.uniprot.org/uniprotkb/Q2H4F5); UPI0001FFF2D9 (https://rest.uniprot.org/uniparc/UPI0001FFF2D9); D5XBA1 (https://rest.uniprot.org/uniprotkb/D5XBA1); UPI000155DF78 (https://rest.uniprot.org/uniparc/UPI000155DF78); D1ZL26 (https://rest.uniprot.org/uniprotkb/D1ZL26); B9K5V6 (https://rest.uniprot.org/uniprotkb/B9K5V6); D6SP58 (https://rest.uniprot.org/uniprotkb/D6SP58); B8KTW7 (https://rest.uniprot.org/uniprotkb/B8KTW7); A1BAB9 (https://rest.uniprot.org/uniprotkb/A1BAB9); E1QPU9 (https://rest.uniprot.org/uniprotkb/E1QPU9); A0LQ66 (https://rest.uniprot.org/uniprotkb/A0LQ66); C8W3B0 (https://rest.uniprot.org/uniprotkb/C8W3B0); A5VNY3 (https://rest.uniprot.org/uniprotkb/A5VNY3); F2L976 (https://rest.uniprot.org/uniprotkb/F2L976); B9XRV7 (https://rest.uniprot.org/uniprotkb/B9XRV7); UPI0001AF585B (https://rest.uniprot.org/uniparc/UPI0001AF585B); F2NMW7 (https://rest.uniprot.org/uniprotkb/F2NMW7); C7MSV3 (https://rest.uniprot.org/uniprotkb/C7MSV3); D4J5N3 (https://rest.uniprot.org/uniprotkb/D4J5N3); B7JYN4 (https://rest.uniprot.org/uniprotkb/B7JYN4); A0LCU2 (https://rest.uniprot.org/uniprotkb/A0LCU2); D3SNV9 (https://rest.uniprot.org/uniprotkb/D3SNV9); A6EZG9 (https://rest.uniprot.org/uniprotkb/A6EZG9); A1AS24 (https://rest.uniprot.org/uniprotkb/A1AS24); A1VFF4 (https://rest.uniprot.org/uniprotkb/A1VFF4); Q1YUS7 (https://rest.uniprot.org/uniprotkb/Q1YUS7); Q02U99 (https://rest.uniprot.org/uniprotkb/Q02U99); C5M662 (https://rest.uniprot.org/uniprotkb/C5M662); C8X4S4 (https://rest.uniprot.org/uniprotkb/C8X4S4); Q4WR52 (https://rest.uniprot.org/uniprotkb/Q4WR52); F0XT90 (https://rest.uniprot.org/uniprotkb/F0XT90); D7WP49 (https://rest.uniprot.org/uniprotkb/D7WP49); E5Y8I7 (https://rest.uniprot.org/uniprotkb/E5Y8I7); A7GQQ7 (https://rest.uniprot.org/uniprotkb/A7GQQ7); F4DUJ5 (https://rest.uniprot.org/uniprotkb/F4DUJ5); D3PDY5 (https://rest.uniprot.org/uniprotkb/D3PDY5); Q9YEU4 (https://rest.uniprot.org/uniprotkb/Q9YEU4); F2DSA8 (https://rest.uniprot.org/uniprotkb/F2DSA8); Q090T7 (https://rest.uniprot.org/uniprotkb/Q090T7); E1V7C1 (https://rest.uniprot.org/uniprotkb/E1V7C1); Q12627 (https://rest.uniprot.org/uniprotkb/Q12627); D8PTG5 (https://rest.uniprot.org/uniprotkb/D8PTG5); D0RQV0 (https://rest.uniprot.org/uniprotkb/D0RQV0); D5X5X3 (https://rest.uniprot.org/uniprotkb/D5X5X3); Q139D3 (https://rest.uniprot.org/uniprotkb/Q139D3); UPI0001854FFB (https://rest.uniprot.org/uniparc/UPI0001854FFB); E8L726 (https://rest.uniprot.org/uniprotkb/E8L726); C5DCP6 (https://rest.uniprot.org/uniprotkb/C5DCP6); C8VH81 (https://rest.uniprot.org/uniprotkb/C8VH81); F2L9W1 (https://rest.uniprot.org/uniprotkb/F2L9W1); B8KU63 (https://rest.uniprot.org/uniprotkb/B8KU63); Q5ARL4 (https://rest.uniprot.org/uniprotkb/Q5ARL4); F2ULL4 (https://rest.uniprot.org/uniprotkb/F2ULL4);

Q0SCE9 (https://rest.uniprot.org/uniprotkb/Q0SCE9); A0JSX6 (https://rest.uniprot.org/uniprotkb/A0JSX6); E6PFZ2 (https://rest.uniprot.org/uniprotkb/E6PFZ2); E3HF45 (https://rest.uniprot.org/uniprotkb/E3HF45); D4YML5 (https://rest.uniprot.org/uniprotkb/D4YML5); E7QRQ9 (https://rest.uniprot.org/uniprotkb/E7QRQ9); F4P8Y5 (https://rest.uniprot.org/uniprotkb/F4P8Y5); F2J298 (https://rest.uniprot.org/uniprotkb/F2J298); C3JTL1 (https://rest.uniprot.org/uniprotkb/C3JTL1); E6WTZ7 (https://rest.uniprot.org/uniprotkb/E6WTZ7); A4YZF5 (https://rest.uniprot.org/uniprotkb/A4YZF5); Q2KZ61 (https://rest.uniprot.org/uniprotkb/Q2KZ61); B2JUT9 (https://rest.uniprot.org/uniprotkb/B2JUT9); A9NWI6 (https://rest.uniprot.org/uniprotkb/A9NWI6); A5DI72 (https://rest.uniprot.org/uniprotkb/A5DI72); B8HCS7 (https://rest.uniprot.org/uniprotkb/B8HCS7); F2N5I2 (https://rest.uniprot.org/uniprotkb/F2N5I2); D3NT19 (https://rest.uniprot.org/uniprotkb/D3NT19); Q4HE83 (https://rest.uniprot.org/uniprotkb/Q4HE83); A7HVW5 (https://rest.uniprot.org/uniprotkb/A7HVW5); Q1QIK8 (https://rest.uniprot.org/uniprotkb/Q1QIK8); D2S425 (https://rest.uniprot.org/uniprotkb/D2S425); D8IRW2 (https://rest.uniprot.org/uniprotkb/D8IRW2); A4WQE7 (https://rest.uniprot.org/uniprotkb/A4WQE7); E0XQ36 (https://rest.uniprot.org/uniprotkb/E0XQ36); B0UAH8 (https://rest.uniprot.org/uniprotkb/B0UAH8); Q7CXZ7 (https://rest.uniprot.org/uniprotkb/Q7CXZ7); B2SZI2 (https://rest.uniprot.org/uniprotkb/B2SZI2); Q4ZLU4 (https://rest.uniprot.org/uniprotkb/Q4ZLU4); A1W0J6 (https://rest.uniprot.org/uniprotkb/A1W0J6); B1L3M9 (https://rest.uniprot.org/uniprotkb/B1L3M9); Q28R08 (https://rest.uniprot.org/uniprotkb/Q28R08); D8IRW4 (https://rest.uniprot.org/uniprotkb/D8IRW4); UPI0000D94179 (https://rest.uniprot.org/uniparc/UPI0000D94179); A9ENW5 (https://rest.uniprot.org/uniprotkb/A9ENW5); A5V4Z2 (https://rest.uniprot.org/uniprotkb/A5V4Z2); D0WQ12 (https://rest.uniprot.org/uniprotkb/D0WQ12); D2L5T6 (https://rest.uniprot.org/uniprotkb/D2L5T6); Q21YE3 (https://rest.uniprot.org/uniprotkb/Q21YE3); B6R8D7 (https://rest.uniprot.org/uniprotkb/B6R8D7); D5G9P7 (https://rest.uniprot.org/uniprotkb/D5G9P7); B8FVI6 (https://rest.uniprot.org/uniprotkb/B8FVI6); D1Z702 (https://rest.uniprot.org/uniprotkb/D1Z702); Q2CIX0 (https://rest.uniprot.org/uniprotkb/Q2CIX0); D5SQP5 (https://rest.uniprot.org/uniprotkb/D5SQP5); B0XXH9 (https://rest.uniprot.org/uniprotkb/B0XXH9); F4AJK4 (https://rest.uniprot.org/uniprotkb/F4AJK4); E8TG55 (https://rest.uniprot.org/uniprotkb/E8TG55); D3T1L7 (https://rest.uniprot.org/uniprotkb/D3T1L7); D3S907 (https://rest.uniprot.org/uniprotkb/D3S907); A4FGG6 (https://rest.uniprot.org/uniprotkb/A4FGG6); D7CMD0 (https://rest.uniprot.org/uniprotkb/D7CMD0); D7A7G4 (https://rest.uniprot.org/uniprotkb/D7A7G4); A9UT16 (https://rest.uniprot.org/uniprotkb/A9UT16); Q0BSS4 (https://rest.uniprot.org/uniprotkb/Q0BSS4); F4CMQ0 (https://rest.uniprot.org/uniprotkb/F4CMQ0); C6BVR4 (https://rest.uniprot.org/uniprotkb/C6BVR4); C6PTU6 (https://rest.uniprot.org/uniprotkb/C6PTU6); Q5FP89 (https://rest.uniprot.org/uniprotkb/Q5FP89); E4NEC2 (https://rest.uniprot.org/uniprotkb/E4NEC2); Q7NCB0 (https://rest.uniprot.org/uniprotkb/Q7NCB0); Q47JF4 (https://rest.uniprot.org/uniprotkb/Q47JF4); Q2KYE1 (https://rest.uniprot.org/uniprotkb/Q2KYE1); D6SLE4 (https://rest.uniprot.org/uniprotkb/D6SLE4); D8F549 (https://rest.uniprot.org/uniprotkb/D8F549); D6ZZG6 (https://rest.uniprot.org/uniprotkb/D6ZZG6); B9WAY2 (https://rest.uniprot.org/uniprotkb/B9WAY2); Q6MJ93 (https://rest.uniprot.org/uniprotkb/Q6MJ93); D1AA97 (https://rest.uniprot.org/uniprotkb/D1AA97); B8MMS5 (https://rest.uniprot.org/uniprotkb/B8MMS5); C4KUH0 (https://rest.uniprot.org/uniprotkb/C4KUH0); F4QMD9 (https://rest.uniprot.org/uniprotkb/F4QMD9); Q6G0E4 (https://rest.uniprot.org/uniprotkb/Q6G0E4); A1CGJ6 (https://rest.uniprot.org/uniprotkb/A1CGJ6); Q6FQM0 (https://rest.uniprot.org/uniprotkb/Q6FQM0); Q6L072 (https://rest.uniprot.org/uniprotkb/Q6L072); C6M397 (https://rest.uniprot.org/uniprotkb/C6M397); A7F310 (https://rest.uniprot.org/uniprotkb/A7F310); B6R001 (https://rest.uniprot.org/uniprotkb/B6R001); C8X203 (https://rest.uniprot.org/uniprotkb/C8X203); Q30TM8 (https://rest.uniprot.org/uniprotkb/Q30TM8); C0S0T8 (https://rest.uniprot.org/uniprotkb/C0S0T8); A6GNK8 (https://rest.uniprot.org/uniprotkb/A6GNK8); C0WB76 (https://rest.uniprot.org/uniprotkb/C0WB76); D8F0T0 (https://rest.uniprot.org/uniprotkb/D8F0T0); D5RHM8 (https://rest.uniprot.org/uniprotkb/D5RHM8); F1QXM5 (https://rest.uniprot.org/uniprotkb/F1QXM5); Q2KG40 (https://rest.uniprot.org/uniprotkb/Q2KG40); D5P7H1 (https://rest.uniprot.org/uniprotkb/D5P7H1); D1AQ90 (https://rest.uniprot.org/uniprotkb/D1AQ90); C7MX80 (https://rest.uniprot.org/uniprotkb/C7MX80); E4M2T1 (https://rest.uniprot.org/uniprotkb/E4M2T1); Q3BUE1 (https://rest.uniprot.org/uniprotkb/Q3BUE1); Q86WU2 (https://rest.uniprot.org/uniprotkb/Q86WU2); E5Y2L5 (https://rest.uniprot.org/uniprotkb/E5Y2L5); A1HP53 (https://rest.uniprot.org/uniprotkb/A1HP53); B4R9H0 (https://rest.uniprot.org/uniprotkb/B4R9H0); D3S2J4 (https://rest.uniprot.org/uniprotkb/D3S2J4); C4YP96 (https://rest.uniprot.org/uniprotkb/C4YP96); B6IU03 (https://rest.uniprot.org/uniprotkb/B6IU03); C7HSG4 (https://rest.uniprot.org/uniprotkb/C7HSG4); Q94AX4 (https://rest.uniprot.org/uniprotkb/Q94AX4); E7RY91 (https://rest.uniprot.org/uniprotkb/E7RY91); B8GBG0 (https://rest.uniprot.org/uniprotkb/B8GBG0); UPI000200186C (https://rest.uniprot.org/uniparc/UPI000200186C); Q5SKY8 (https://rest.uniprot.org/uniprotkb/Q5SKY8); B8FWM1 (https://rest.uniprot.org/uniprotkb/B8FWM1); Q5LMT4 (https://rest.uniprot.org/uniprotkb/Q5LMT4); E0QJI9 (https://rest.uniprot.org/uniprotkb/E0QJI9); D2UA72 (https://rest.uniprot.org/uniprotkb/D2UA72); A9FEI8 (https://rest.uniprot.org/uniprotkb/A9FEI8); B9J9W4 (https://rest.uniprot.org/uniprotkb/B9J9W4); A6G2T7 (https://rest.uniprot.org/uniprotkb/A6G2T7); A5VH95 (https://rest.uniprot.org/uniprotkb/A5VH95); F0LDK1 (https://rest.uniprot.org/uniprotkb/F0LDK1); B6R417 (https://rest.uniprot.org/uniprotkb/B6R417); B3DUE7 (https://rest.uniprot.org/uniprotkb/B3DUE7); C8XJR7 (https://rest.uniprot.org/uniprotkb/C8XJR7);

E1VGQ6 (https://rest.uniprot.org/uniprotkb/E1VGQ6); B9R0G5 (https://rest.uniprot.org/uniprotkb/B9R0G5); UPI00016A8049 (https://rest.uniprot.org/uniparc/UPI00016A8049); E7R370 (https://rest.uniprot.org/uniprotkb/E7R370); UPI00020EF04B (https://rest.uniprot.org/uniparc/UPI00020EF04B); Q0U9Y3 (https://rest.uniprot.org/uniprotkb/Q0U9Y3); Q2G8T9 (https://rest.uniprot.org/uniprotkb/Q2G8T9); A3TY45 (https://rest.uniprot.org/uniprotkb/A3TY45); A9RFK0 (https://rest.uniprot.org/uniprotkb/A9RFK0); B2IEC5 (https://rest.uniprot.org/uniprotkb/B2IEC5); Q31RL1 (https://rest.uniprot.org/uniprotkb/Q31RL1); Q16CG8 (https://rest.uniprot.org/uniprotkb/Q16CG8); Q2RWB4 (https://rest.uniprot.org/uniprotkb/Q2RWB4); B2UBS3 (https://rest.uniprot.org/uniprotkb/B2UBS3); E2RSL6 (https://rest.uniprot.org/uniprotkb/E2RSL6); B2WBK4 (https://rest.uniprot.org/uniprotkb/B2WBK4); UPI0000E49631 (https://rest.uniprot.org/uniparc/UPI0000E49631); A1RZY9 (https://rest.uniprot.org/uniprotkb/A1RZY9); F2L975 (https://rest.uniprot.org/uniprotkb/F2L975); B3PR95 (https://rest.uniprot.org/uniprotkb/B3PR95); F2ZIS8 (https://rest.uniprot.org/uniprotkb/F2ZIS8); A4Z0V5 (https://rest.uniprot.org/uniprotkb/A4Z0V5); A9IQN3 (https://rest.uniprot.org/uniprotkb/A9IQN3); D6WE92 (https://rest.uniprot.org/uniprotkb/D6WE92); A8TMB9 (https://rest.uniprot.org/uniprotkb/A8TMB9); D7E531 (https://rest.uniprot.org/uniprotkb/D7E531); B6HF53 (https://rest.uniprot.org/uniprotkb/B6HF53); B2A9U2 (https://rest.uniprot.org/uniprotkb/B2A9U2); A0B9P4 (https://rest.uniprot.org/uniprotkb/A0B9P4); Q5LVR6 (https://rest.uniprot.org/uniprotkb/Q5LVR6); A3SN13 (https://rest.uniprot.org/uniprotkb/A3SN13); Q48GS1 (https://rest.uniprot.org/uniprotkb/Q48GS1); D9MP61 (https://rest.uniprot.org/uniprotkb/D9MP61); B6JFL6 (https://rest.uniprot.org/uniprotkb/B6JFL6); C3MEF7 (https://rest.uniprot.org/uniprotkb/C3MEF7); E4VHS6 (https://rest.uniprot.org/uniprotkb/E4VHS6); E1FPX3 (https://rest.uniprot.org/uniprotkb/E1FPX3); A7HEH5 (https://rest.uniprot.org/uniprotkb/A7HEH5); F3KWW8 (https://rest.uniprot.org/uniprotkb/F3KWW8); B7A6Q4 (https://rest.uniprot.org/uniprotkb/B7A6Q4); D3PKP2 (https://rest.uniprot.org/uniprotkb/D3PKP2); B3QCS9 (https://rest.uniprot.org/uniprotkb/B3QCS9); F4GQX1 (https://rest.uniprot.org/uniprotkb/F4GQX1); Q47CF1 (https://rest.uniprot.org/uniprotkb/Q47CF1); D2S485 (https://rest.uniprot.org/uniprotkb/D2S485); D0CRN4 (https://rest.uniprot.org/uniprotkb/D0CRN4); F0NTR0 (https://rest.uniprot.org/uniprotkb/F0NTR0); D4KDA2 (https://rest.uniprot.org/uniprotkb/D4KDA2); A5UV62 (https://rest.uniprot.org/uniprotkb/A5UV62); A1WTK3 (https://rest.uniprot.org/uniprotkb/A1WTK3); A7IF77 (https://rest.uniprot.org/uniprotkb/A7IF77); D0MVU6 (https://rest.uniprot.org/uniprotkb/D0MVU6); C7RC96 (https://rest.uniprot.org/uniprotkb/C7RC96); B3TCR6 (https://rest.uniprot.org/uniprotkb/B3TCR6); E1TIM9 (https://rest.uniprot.org/uniprotkb/E1TIM9); D3SVG0 (https://rest.uniprot.org/uniprotkb/D3SVG0); E5WCF3 (https://rest.uniprot.org/uniprotkb/E5WCF3); B7V5P6 (https://rest.uniprot.org/uniprotkb/B7V5P6); A6GRB5 (https://rest.uniprot.org/uniprotkb/A6GRB5); F4B7T1 (https://rest.uniprot.org/uniprotkb/F4B7T1); B6AWL9 (https://rest.uniprot.org/uniprotkb/B6AWL9); C1G809 (https://rest.uniprot.org/uniprotkb/C1G809); F0XU52 (https://rest.uniprot.org/uniprotkb/F0XU52); A6CMI5 (https://rest.uniprot.org/uniprotkb/A6CMI5); D5QT43 (https://rest.uniprot.org/uniprotkb/D5QT43); Q5P348 (https://rest.uniprot.org/uniprotkb/Q5P348); A6E466 (https://rest.uniprot.org/uniprotkb/A6E466); F2KJ62 (https://rest.uniprot.org/uniprotkb/F2KJ62); Q89GX1 (https://rest.uniprot.org/uniprotkb/Q89GX1); F0SKG1 (https://rest.uniprot.org/uniprotkb/F0SKG1); B8IND4 (https://rest.uniprot.org/uniprotkb/B8IND4); Q0FDJ1 (https://rest.uniprot.org/uniprotkb/Q0FDJ1); B2JGF8 (https://rest.uniprot.org/uniprotkb/B2JGF8); E7REF4 (https://rest.uniprot.org/uniprotkb/E7REF4); C6CFL7 (https://rest.uniprot.org/uniprotkb/C6CFL7); C7ZG87 (https://rest.uniprot.org/uniprotkb/C7ZG87); B2UGL8 (https://rest.uniprot.org/uniprotkb/B2UGL8); A3V757 (https://rest.uniprot.org/uniprotkb/A3V757); B2IV23 (https://rest.uniprot.org/uniprotkb/B2IV23); C0QJA3 (https://rest.uniprot.org/uniprotkb/C0QJA3); C3AUF3 (https://rest.uniprot.org/uniprotkb/C3AUF3); Q3SP41 (https://rest.uniprot.org/uniprotkb/Q3SP41); A5WAL9 (https://rest.uniprot.org/uniprotkb/A5WAL9); Q97B62 (https://rest.uniprot.org/uniprotkb/Q97B62); D1CHX7 (https://rest.uniprot.org/uniprotkb/D1CHX7); E6LB87 (https://rest.uniprot.org/uniprotkb/E6LB87); D5CPC5 (https://rest.uniprot.org/uniprotkb/D5CPC5); C3YFC6 (https://rest.uniprot.org/uniprotkb/C3YFC6); F4Q4J3 (https://rest.uniprot.org/uniprotkb/F4Q4J3); A5DUQ9 (https://rest.uniprot.org/uniprotkb/A5DUQ9); A7IGH2 (https://rest.uniprot.org/uniprotkb/A7IGH2); E5A1Y4 (https://rest.uniprot.org/uniprotkb/E5A1Y4); E5WGB3 (https://rest.uniprot.org/uniprotkb/E5WGB3); Q3IPH8 (https://rest.uniprot.org/uniprotkb/Q3IPH8); D5V0Z5 (https://rest.uniprot.org/uniprotkb/D5V0Z5); A1VGJ4 (https://rest.uniprot.org/uniprotkb/A1VGJ4); A6QC61 (https://rest.uniprot.org/uniprotkb/A6QC61); D0N339 (https://rest.uniprot.org/uniprotkb/D0N339); A0R298 (https://rest.uniprot.org/uniprotkb/A0R298); A6EJW9 (https://rest.uniprot.org/uniprotkb/A6EJW9); A9WF29 (https://rest.uniprot.org/uniprotkb/A9WF29); A4EQ89 (https://rest.uniprot.org/uniprotkb/A4EQ89); D9SZH5 (https://rest.uniprot.org/uniprotkb/D9SZH5); F0RUG4 (https://rest.uniprot.org/uniprotkb/F0RUG4); A1L2R0 (https://rest.uniprot.org/uniprotkb/A1L2R0); A1URV6 (https://rest.uniprot.org/uniprotkb/A1URV6); A3W2Z7 (https://rest.uniprot.org/uniprotkb/A3W2Z7); F3LMV7 (https://rest.uniprot.org/uniprotkb/F3LMV7); B8DRH5 (https://rest.uniprot.org/uniprotkb/B8DRH5); Q47AT6 (https://rest.uniprot.org/uniprotkb/Q47AT6); C7YMF9 (https://rest.uniprot.org/uniprotkb/C7YMF9); Q0ABX5 (https://rest.uniprot.org/uniprotkb/Q0ABX5); UPI00016A825E (https://rest.uniprot.org/uniparc/UPI00016A825E); B9JSJ4 (https://rest.uniprot.org/uniprotkb/B9JSJ4); A8LIY9 (https://rest.uniprot.org/uniprotkb/A8LIY9); E9EVP9 (https://rest.uniprot.org/uniprotkb/E9EVP9); B9YZ18

(https://rest.uniprot.org/uniprotkb/B9YZ18); B9D2T3 (https://rest.uniprot.org/uniprotkb/B9D2T3); UPI000190836F (https://rest.uniprot.org/uniparc/UPI000190836F); E4L019 (https://rest.uniprot.org/uniprotkb/E4L019); B9MG03 (https://rest.uniprot.org/uniprotkb/B9MG03); A9KNB3 (https://rest.uniprot.org/uniprotkb/A9KNB3); E4TWJ4 (https://rest.uniprot.org/uniprotkb/E4TWJ4); A6WZS9 (https://rest.uniprot.org/uniprotkb/A6WZS9); A8ZRP5 (https://rest.uniprot.org/uniprotkb/A8ZRP5); Q6G4E1 (https://rest.uniprot.org/uniprotkb/Q6G4E1); UPI0001CBAFD0 (https://rest.uniprot.org/uniparc/UPI0001CBAFD0); Q1QU24 (https://rest.uniprot.org/uniprotkb/Q1QU24); C8Q182 (https://rest.uniprot.org/uniprotkb/C8Q182); A9I727 (https://rest.uniprot.org/uniprotkb/A9I727); Q1MY43 (https://rest.uniprot.org/uniprotkb/Q1MY43); F4LBJ2 (https://rest.uniprot.org/uniprotkb/F4LBJ2); Q2GA62 (https://rest.uniprot.org/uniprotkb/Q2GA62); C0NLP1 (https://rest.uniprot.org/uniprotkb/C0NLP1); Q2KXD0 (https://rest.uniprot.org/uniprotkb/Q2KXD0); D4XF63 (https://rest.uniprot.org/uniprotkb/D4XF63); C8WT16 (https://rest.uniprot.org/uniprotkb/C8WT16); D3UGC4 (https://rest.uniprot.org/uniprotkb/D3UGC4); C7QF73 (https://rest.uniprot.org/uniprotkb/C7QF73); A9C0T3 (https://rest.uniprot.org/uniprotkb/A9C0T3); Q3JLQ7 (https://rest.uniprot.org/uniprotkb/Q3JLQ7); Q4PFP4 (https://rest.uniprot.org/uniprotkb/Q4PFP4); C0EX13 (https://rest.uniprot.org/uniprotkb/C0EX13); A3LTY9 (https://rest.uniprot.org/uniprotkb/A3LTY9); A6UB34 (https://rest.uniprot.org/uniprotkb/A6UB34); D8J206 (https://rest.uniprot.org/uniprotkb/D8J206); D9URI3 (https://rest.uniprot.org/uniprotkb/D9URI3); A8IJ56 (https://rest.uniprot.org/uniprotkb/A8IJ56); F2THR3 (https://rest.uniprot.org/uniprotkb/F2THR3); B8FFL9 (https://rest.uniprot.org/uniprotkb/B8FFL9); A1U5V6 (https://rest.uniprot.org/uniprotkb/A1U5V6); Q10XC5 (https://rest.uniprot.org/uniprotkb/Q10XC5); B3QHP8 (https://rest.uniprot.org/uniprotkb/B3QHP8); A1CFY7 (https://rest.uniprot.org/uniprotkb/A1CFY7); C3KC25 (https://rest.uniprot.org/uniprotkb/C3KC25); A3Z1I2 (https://rest.uniprot.org/uniprotkb/A3Z1I2); C5GN98 (https://rest.uniprot.org/uniprotkb/C5GN98); F0UJP7 (https://rest.uniprot.org/uniprotkb/F0UJP7); B7K3E0 (https://rest.uniprot.org/uniprotkb/B7K3E0); C0Z8J6 (https://rest.uniprot.org/uniprotkb/C0Z8J6); Q2SA67 (https://rest.uniprot.org/uniprotkb/Q2SA67); Q1YGJ9 (https://rest.uniprot.org/uniprotkb/Q1YGJ9); Q13BQ5 (https://rest.uniprot.org/uniprotkb/Q13BQ5); Q11GU7 (https://rest.uniprot.org/uniprotkb/Q11GU7); D5TT74 (https://rest.uniprot.org/uniprotkb/D5TT74); UPI0001B45946 (https://rest.uniprot.org/uniparc/UPI0001B45946); F4GW59 (https://rest.uniprot.org/uniprotkb/F4GW59); UPI00020D3CC7 (https://rest.uniprot.org/uniparc/UPI00020D3CC7); Q7MAG6 (https://rest.uniprot.org/uniprotkb/Q7MAG6); UPI0001745B2A (https://rest.uniprot.org/uniparc/UPI0001745B2A); D5RJX5 (https://rest.uniprot.org/uniprotkb/D5RJX5); B3S947 (https://rest.uniprot.org/uniprotkb/B3S947); E0K0U2 (https://rest.uniprot.org/uniprotkb/E0K0U2); Q8TH91 (https://rest.uniprot.org/uniprotkb/Q8TH91); B8KLB5 (https://rest.uniprot.org/uniprotkb/B8KLB5); F3L1Z9 (https://rest.uniprot.org/uniprotkb/F3L1Z9); P39976 (https://rest.uniprot.org/uniprotkb/P39976); UPI000180BEFF (https://rest.uniprot.org/uniparc/UPI000180BEFF); Q2IYH6 (https://rest.uniprot.org/uniprotkb/Q2IYH6); C5PB22 (https://rest.uniprot.org/uniprotkb/C5PB22); E6VT36 (https://rest.uniprot.org/uniprotkb/E6VT36); D5APN1 (https://rest.uniprot.org/uniprotkb/D5APN1); C7LQT0 (https://rest.uniprot.org/uniprotkb/C7LQT0); Q0G645 (https://rest.uniprot.org/uniprotkb/Q0G645); D8F1U2 (https://rest.uniprot.org/uniprotkb/D8F1U2); A1B435 (https://rest.uniprot.org/uniprotkb/A1B435); Q0FDC9 (https://rest.uniprot.org/uniprotkb/Q0FDC9); A9IQL6 (https://rest.uniprot.org/uniprotkb/A9IQL6); A7SQV7 (https://rest.uniprot.org/uniprotkb/A7SQV7); Q0A7E9 (https://rest.uniprot.org/uniprotkb/Q0A7E9); D3FW49 (https://rest.uniprot.org/uniprotkb/D3FW49); C6BD57 (https://rest.uniprot.org/uniprotkb/C6BD57); Q0C363 (https://rest.uniprot.org/uniprotkb/Q0C363); D8QSH8 (https://rest.uniprot.org/uniprotkb/D8QSH8); D8PKA6 (https://rest.uniprot.org/uniprotkb/D8PKA6); D4TDU2 (https://rest.uniprot.org/uniprotkb/D4TDU2); F4XKR7 (https://rest.uniprot.org/uniprotkb/F4XKR7);

Supplementary Table 1. Distribution of mutants selected for thermostability according to their final score after visual inspection.

**Final score**	0	1	2	3	4	n/a
**Number of mutants**	72	185	185	45	4	5

As described in the experimental procedures, the mutants were assessed based on the physicochemical interactions between the new residues and their environment and the structural variability. A score of 1 was assigned to every detrimental effect observed per mutation. 72 mutations scored 0 and were selected for further analysis. In the last column (n/a), the number of mutants for which MD failed is listed.

Supplementary Table 2. Kinetic and thermostability properties of EUGO variants.

Variants	T_m (°C)	Δ T_m (°C)	K_M (μM) [a]	k_{cat} (s^{-1})[a]
Wild-type EUGO	66.5 ± 0.0	-	43.0 ± 2.2	6.8 ± 0.2
H434Y-EUGO	73.0 ± 0.4	+6.5	142.9 ± 12.6	2.7 ± 0.1
H434W-EUGO	70.0 ± 0.0	+3.5	124.5 ± 9.1	1.9 ± 0.1
S81H-EUGO	71.2 ± 0.3	+4.7	44.8 ± 3.4	4.8 ± 0.1
A423M-EUGO	69.2 ± 0.3	+2.7	75.4 ± 3.6	5.3 ± 0.1
S518P-EUGO	69.0 ± 0.0	+2.5	37.6 ± 2.5	4.6 ± 0.1
D202N-EUGO	69.0 ± 0.7	+2.5	7.4 ± 0.3	1.3 ± 0.0
I445D-EUGO	68.5 ± 0.0	+2.0	65.0 ± 6.0	5.7 ± 0.1
EUGO2X (S81H+A423M)	72.0 ± 0.0	+5.5	118.2 ± 10.9	6.8 ± 0.2
EUGO3X (S81H+A423M+ S518P)	73.5 ± 0.0	+7.0	73.1 ± 5.0	5.8 ± 0.2
EUGO4X (S81H+A423M+S518P+I445D)	74.0 ± 0.0	+7.5	92 ± 10.8	6.5 ± 0.3
EUGO5X (S81H+A423M+S518P+I445D+H434Y)	80.0 ± 0.0	+13.5	177.6 ± 15.8	4.8 ± 0.2

[a] K_M and k_{cat} values are based on rates determined at 7 different substrate concentrations (each in duplicate); for each average, the error was smaller than 5%. Vanillyl alcohol was used as a substrate.

Supplementary Table 3. Data collection and refinement statistics for S394V-EUGO5X and PROGO.

	S394V-EUGO5X	***PROGO***
Space group	$P2_1$	I2
Unit cell axes (Å)	149.64, 79.43, 189.18	114.19, 142.40, 288.52
Unit cell angles (°)	90, 96.25, 90	90, 100.41, 90
Resolution (Å)	2.8	2.4
PDB code	7YWU	7YWV
$R_{sym}{}^{a,b}$	0.166 (1.099)	0.129 (1.264)
$CC_{1/2}$	0.974 (0.468)	0.992 (0.466)
Completeness^{b} (%)	97.8 (98.8)	99.3 (100.0)
Unique reflections	106726	175685
Redundancy	3.5 (3.7)	3.1 (3.2)
I/σ^{b}	6.6 (1.3)	7.8 (1.1)
N° of non-hydrogen atoms	32569/8x53	33568/8x53
protein/FAD	8x13	8x13
ligand	33	313
water		
Average B value (Å^2)	55.77	58.80
$R_{crys}{}^{c}$ (%)	22.9	22.23
$R_{free}{}^{c}$ (%)	27.4	26.16
Rms bond length (Å)	0.014	0.014
Rms bond angles (°)	1.81	1.77

^{a} $R_{sym}=\sum|I_i-\langle I\rangle|/\sum I_i$, where I_i is the intensity of ith observation and <I> is the mean intensity of the reflection.

^{b} Values in parentheses are for reflections in the highest resolution shell.

^{c} $R_{cryst}=\sum|F_{obs}-F_{calc}|/\sum|F_{obs}|$ where F_{obs} and F_{calc} are the observed and calculated structure factor amplitudes, respectively. R_{cryst} and R_{free} were calculated using the working and test sets, respectively.

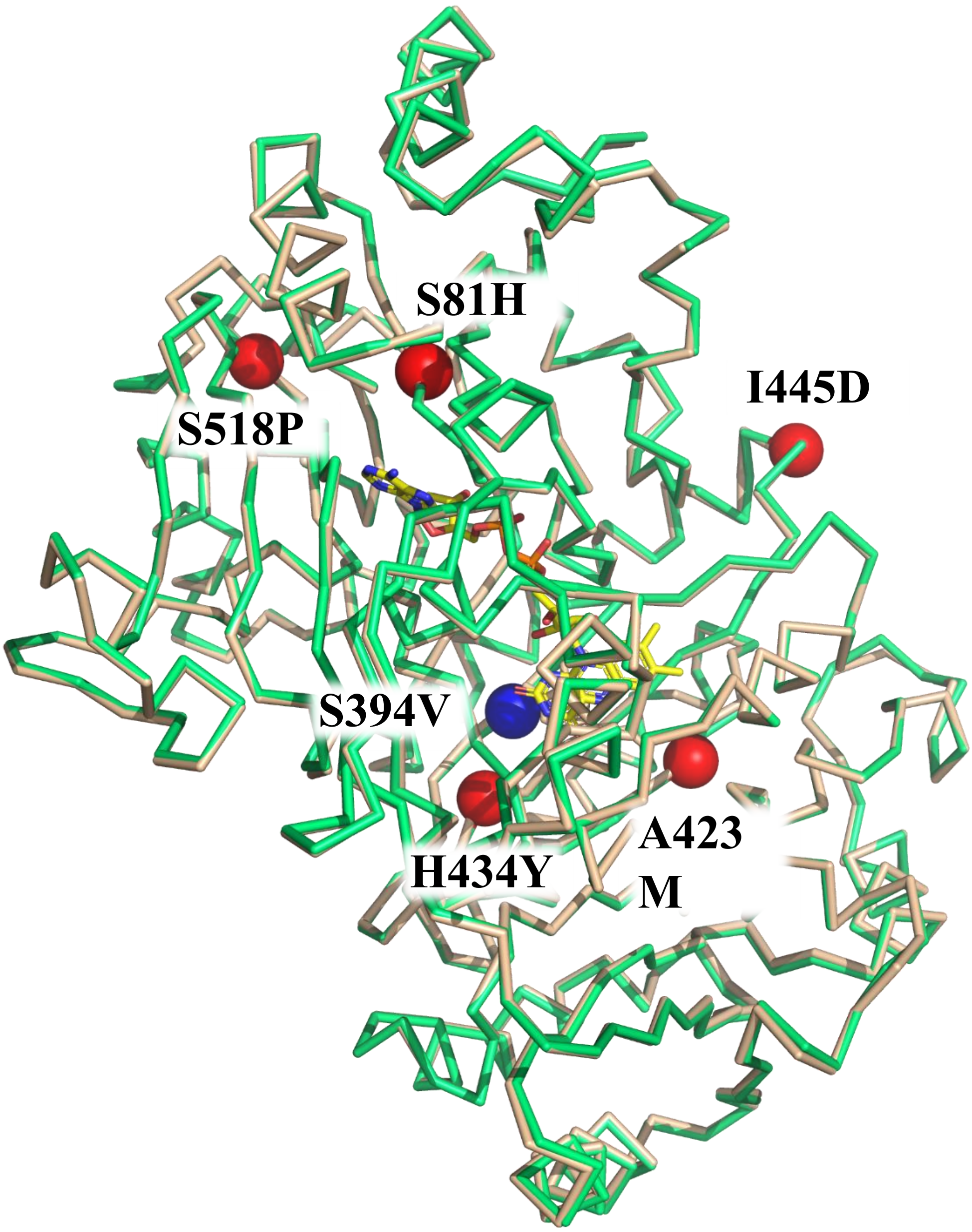

Supplementary Figure 1. Comparison between wild-type EUGO and S394V-EUGO5X. Superposition of the backbone of wild-type EUGO (wheat) and S394V-EUGO5X (green). The five stabilizing mutation sites are represented with red spheres. The chemoselectivity-affording S394V mutation is indicated as a blue sphere. The FAD is shown in yellow sticks.

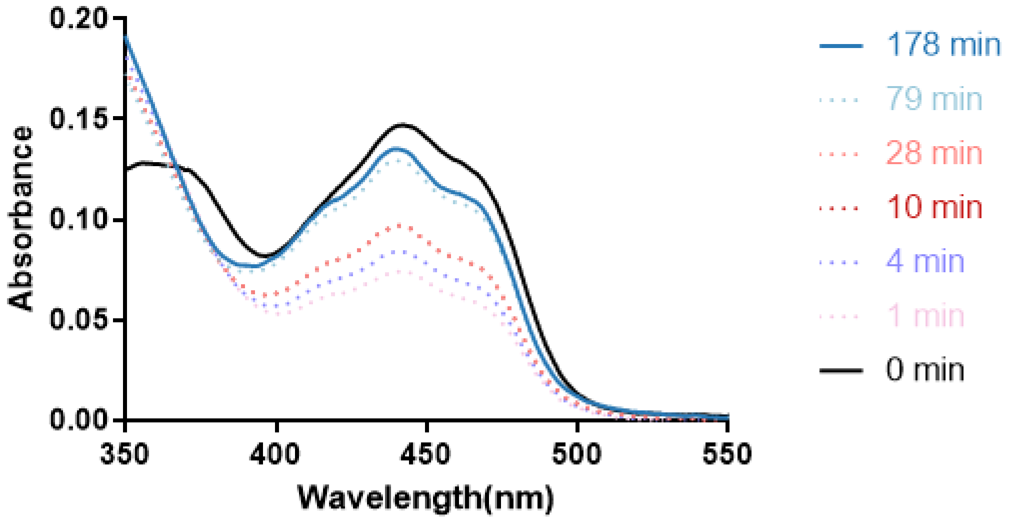

Supplementary Figure 2. Time-course absorbance spectra of an incubation of 200 µM 4-n-propylguaiacol with 10 µM S394V-EUGO5X (50 mM KPi, pH 7.5, 25°C). Absorbance spectra were collected at indicated time intervals. The fully oxidized enzyme (t = 0) is also included (black line) by collecting the absorbance spectrum before addition of substrate. Upon adding the substrate, the absorbance of the flavin cofactor at around 450 nm is largely reduced, indicative for a partial reduced or modified flavin cofactor. At the end of the incubation, conversion of 4-n-propylguaiacol is complete and the oxidized flavin cofactor is restored.

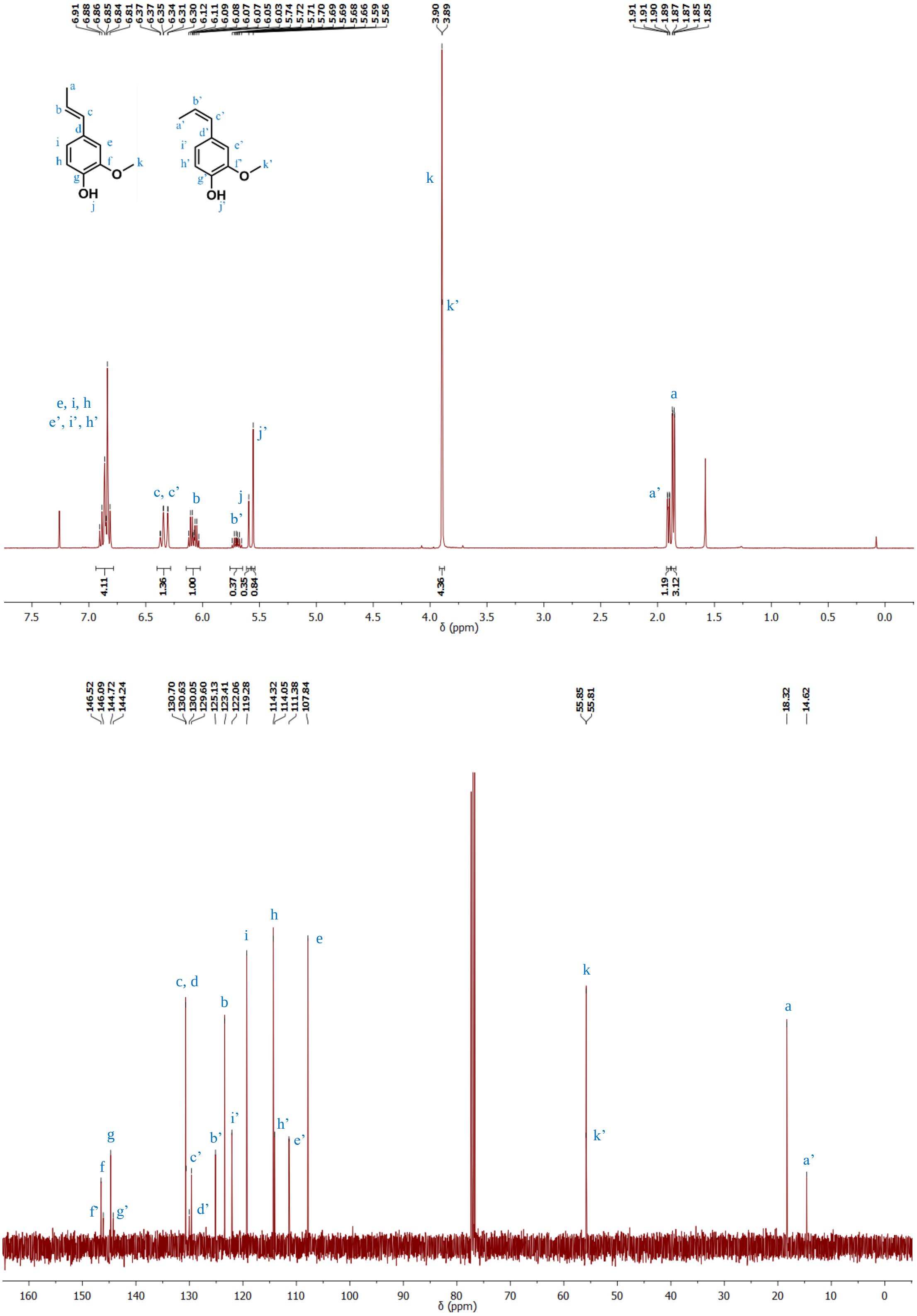

Supplementary Figure 3. ^{1}H NMR (top) and ^{13}C (bottom) spectra of E/Z isoeugenol isolated from whole-cells catalyzed reaction.
