# Supplementary material for: Structure- and computational-aided engineering of an oxidase to produce isoeugenol from a lignin-derived compound
Source: Nat Commun. 2022 Nov 23;13:7195. doi: 10.1038/s41467-022-34912-3 (PMC9684555; doi:10.1038/s41467-022-34912-3)
Supplement: Supplementary file 3 — Reporting Summary [file 41467_2022_34912_MOESM3_ESM.pdf]

nature portfolio

Corresponding author(s): Marco Fraaije
Last updated by author(s): 2022-11-03

Reporting Summary

Nature Portfolio wishes to improve the reproducibility of the work that we publish. This form provides structure for consistency and transparency in reporting. For further information on Nature Portfolio policies, see our Editorial Policies and the Editorial Policy Checklist.

Statistics

For all statistical analyses, confirm that the following items are present in the figure legend, table legend, main text, or Methods section.

n/a	Confirmed	
☐	☒	The exact sample size (*n*) for each experimental group/condition, given as a discrete number and unit of measurement
☒	☐	A statement on whether measurements were taken from distinct samples or whether the same sample was measured repeatedly
☒	☐	The statistical test(s) used AND whether they are one- or two-sided<br>*Only common tests should be described solely by name; describe more complex techniques in the Methods section.*
☒	☐	A description of all covariates tested
☒	☐	A description of any assumptions or corrections, such as tests of normality and adjustment for multiple comparisons
☐	☒	A full description of the statistical parameters including central tendency (e.g. means) or other basic estimates (e.g. regression coefficient) AND variation (e.g. standard deviation) or associated estimates of uncertainty (e.g. confidence intervals)
☒	☐	For null hypothesis testing, the test statistic (e.g. *F*, *t*, *r*) with confidence intervals, effect sizes, degrees of freedom and *P* value noted<br>*Give P values as exact values whenever suitable.*
☒	☐	For Bayesian analysis, information on the choice of priors and Markov chain Monte Carlo settings
☒	☐	For hierarchical and complex designs, identification of the appropriate level for tests and full reporting of outcomes
☒	☐	Estimates of effect sizes (e.g. Cohen's *d*, Pearson's *r*), indicating how they were calculated

Our web collection on statistics for biologists contains articles on many of the points above.

Software and code

Policy information about availability of computer code

Data collection	No code was used. Data collection for structure determination was done at the indicated synchrotron facilities (PXI beamline of the Swiss Light Source in Villigen (SLS, Switzerland) and at Massif3 beamline of the European Synchrotron Radiation Facility (ESRF, Grenoble).
Data analysis	Crystallographic data handling was performed with COOT and Phenix and figures were created with PyMOL (DeLano Scientific; www.pymol.org) and Chimera. The exact versions were: COOT-0.8.9.2, PyMol-2.4, Chimera-1.16, Phenix-1.20.1

For manuscripts utilizing custom algorithms or software that are central to the research but not yet described in published literature, software must be made available to editors and reviewers. We strongly encourage code deposition in a community repository (e.g. GitHub). See the Nature Portfolio guidelines for submitting code & software for further information.

Data

Policy information about availability of data

All manuscripts must include a data availability statement. This statement should provide the following information, where applicable:

- Accession codes, unique identifiers, or web links for publicly available datasets
- A description of any restrictions on data availability
- For clinical datasets or third party data, please ensure that the statement adheres to our policy

The structural coordinates generated in this study have been deposited in the Protein Data Bank under accession codes: 7YWU and 7YWV. The structural coordinates of wild-type EUGO (5FXD) are available from the Protein Data Bank. All data and materials supporting the findings in the manuscript are available in the Supplementary Information and the Source Data file and from the corresponding author upon request.

Human research participants

Policy information about studies involving human research participants and Sex and Gender in Research.

Reporting on sex and gender	n.a.
Population characteristics	n.a.
Recruitment	n.a.
Ethics oversight	n.a.

Note that full information on the approval of the study protocol must also be provided in the manuscript.

Field-specific reporting

Please select the one below that is the best fit for your research. If you are not sure, read the appropriate sections before making your selection.

☒ Life sciences ☐ Behavioural & social sciences ☐ Ecological, evolutionary & environmental sciences

For a reference copy of the document with all sections, see nature.com/documents/nr-reporting-summary-flat.pdf

Life sciences study design

All studies must disclose on these points even when the disclosure is negative.

Sample size	Sample size was determined based on previous publications on similar experiments. No statistical methods were used to predetermine sample size.
Data exclusions	No data was excluded.
Replication	Experiments (enzyme production, purification, kinetic analyses and conversions) were done in duplicate and found to be reproducible.
Randomization	Enzyme mutants were individually prepared and treated identical, therefore no randomization was performed.
Blinding	Enzyme mutants were individually prepared and treated identical, no blinding was done.

Reporting for specific materials, systems and methods

We require information from authors about some types of materials, experimental systems and methods used in many studies. Here, indicate whether each material, system or method listed is relevant to your study. If you are not sure if a list item applies to your research, read the appropriate section before selecting a response.

Materials & experimental systems

n/a	Involved in the study	
☒	☐	Antibodies
☒	☐	Eukaryotic cell lines
☒	☐	Palaeontology and archaeology
☒	☐	Animals and other organisms
☒	☐	Clinical data
☒	☐	Dual use research of concern

Methods

n/a	Involved in the study	
☒	☐	ChIP-seq
☒	☐	Flow cytometry
☒	☐	MRI-based neuroimaging

Animals and other research organisms

Policy information about studies involving animals; ARRIVE guidelines recommended for reporting animal research, and Sex and Gender in Research

Laboratory animals	n.a.
Wild animals	n.a.

Reporting on sex	n.a.
Field-collected samples	n.a.
Ethics oversight	n.a.

Note that full information on the approval of the study protocol must also be provided in the manuscript.
